# Discovery and characterization of a novel class of cyclic peptidic compounds inhibiting the subunit interaction of the protein kinase CK2α_2_β_2_ holoenzyme

**DOI:** 10.1039/d6cb00095a

**Published:** 2026-06-23

**Authors:** Christian Werner, Sophia Eimermacher, Miriam Lauwers, Dirk Lindenblatt, Esra Seymen, Ekaterina Kulko, Leonard Klein, Michaela Steinkrüger, Robin Baumann, Sarah Salamon, Cora Fried, Dietmar Fischer, Andreas Oder, Martin Neuenschwander, Claudia Götz, Karsten Niefind, Markus Pietsch

**Affiliations:** a Universität zu Köln, Department für Chemie und Biochemie, Institut für Biochemie, Zülpicher Straße 47 D-50674 Köln Germany karsten.niefind@uni-koeln.de; b Technische Hochschule Köln, Fakultät für Angewandte Naturwissenschaften, Campusplatz 1 D-51379 Leverkusen Germany markus.pietsch@th-koeln.de; c Universität zu Köln, Medizinische Fakultät und Uniklinik Köln, Zentrum für Pharmakologie, Institute I&II für Pharmakologie, Gleueler Straße 24 D-50931 Köln Germany; d Leibniz-Forschungsinstitut für Molekulare Pharmakologie im Forschungsverbund Berlin e.V. (FMP), Screening Unit, Campus Berlin-Buch, Robert-Roessle-Straße 10 D-13125 Berlin Germany; e Universität des Saarlandes, Medizinische Biochemie und Molekularbiologie, Kirrbergerstraße, Gebäude 44 D-66421 Homburg Germany

## Abstract

CK2 belongs to the eukaryotic protein kinase superfamily and has a distinctive heterotetrameric quaternary structure. In the so-called CK2α_2_β_2_ holoenzyme, which is the main form of CK2 in human cells, two catalytic subunits (CK2α) are attached to a stable homodimer of non-catalytic subunits (CK2β). Most CK2 inhibitors described so far are ATP-competitive. Some of them are the subject of clinical studies because the enzyme is upregulated in many tumours, and CK2 activity contributes to tumour survival. An unconventional method to interfere with CK2 is to disturb the CK2α/CK2β interaction with small molecules. In a high-throughput screen against a 67 000-compound library using a fluorescence anisotropy-based displacement assay, we identified a group of derivatised cyclic pentapeptides that target the CK2β binding site of CK2α. The CK2β-competitive effect of these screening hits was verified using either human CK2α or its paralogous isoenzyme CK2α′ as interaction partners. To this end, novel Förster resonance energy transfer-based CK2α/CK2β and CK2α′/CK2β interaction assays were developed and extensively tested. A result of these validation measurements was that the CK2β-antagonists bind more strongly to CK2α than to CK2α′ although both paralogs do not differ in the amino acid composition at their CK2β interfaces. Co-crystallisation experiments of two high-affinity binders with CK2α and CK2α′ led to three complex structures that confirm the binding of the compounds to the CK2β interface. The structures suggest a critical role of the β4β5-loop for the higher affinity of the compounds to CK2α compared to CK2α′. Furthermore, they offer suggestions on how to enhance their efficacy in the future.

## Abstract 2

A high-throughput screening led to the discovery of cyclic pentapeptides binding to the CK2β-site of both CK2α and CK2α′. These compounds disturb the assembly of both CK2α_2_β_2_ and CK2α′_2_β_2_ holoenzymes and induce holoenzyme dissociation.

## Graphical abstract

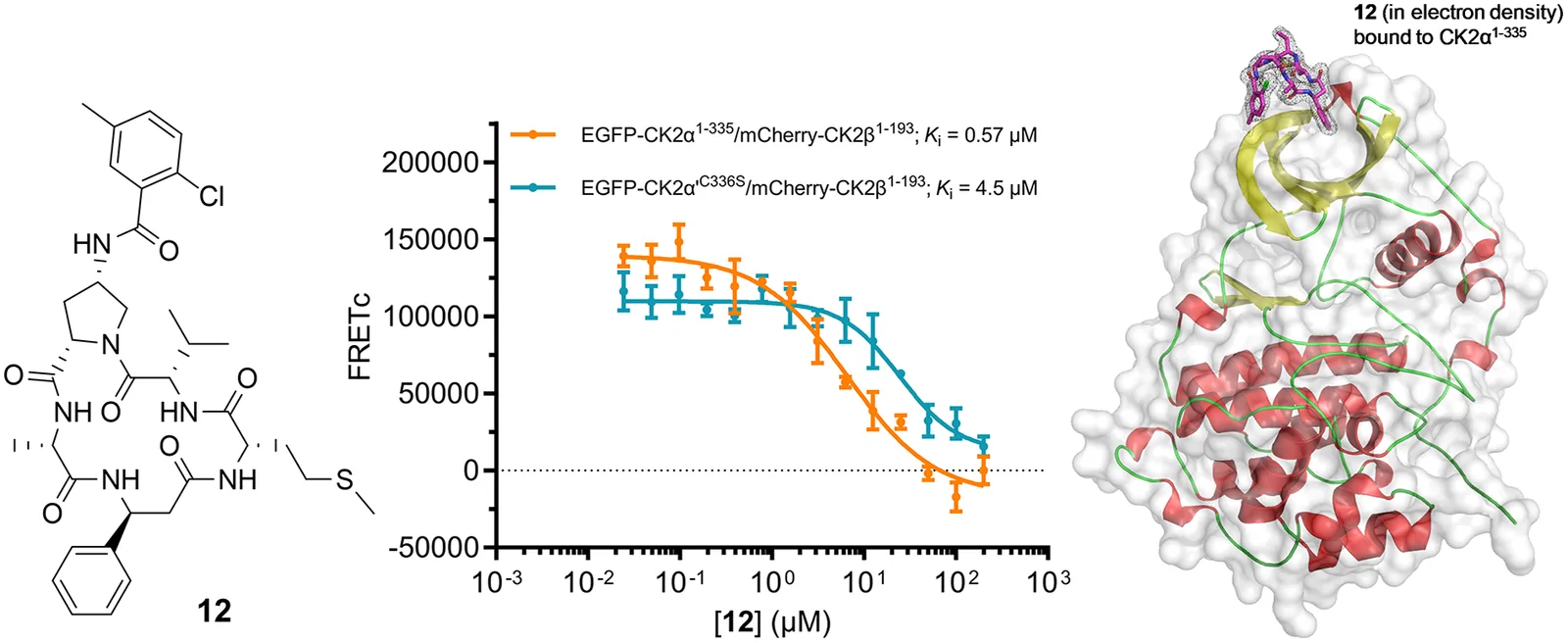

## Introduction

**Fig. 1:**
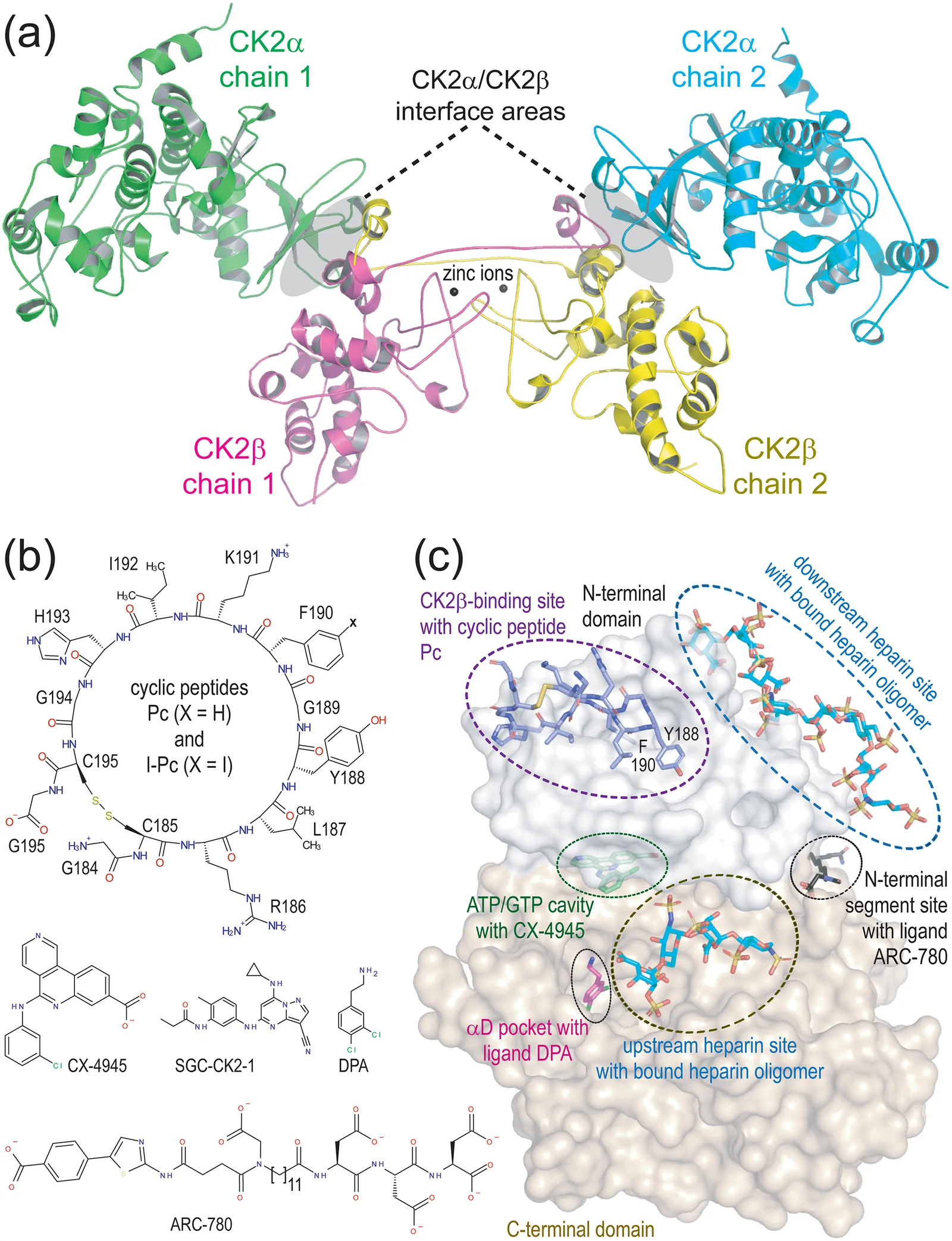
Structure and ligand binding of protein kinase CK2. (a) Architecture of the heterotetrameric CK2α_2_β_2_ holoenzyme.^22^ (b) Selected artificial CK2α or CK2α′ ligands mentioned in this work; the sequence numbering of the cyclic peptide Pc is derived from human CK2β; 1,3-dichlorophenethylamine (DPA) and ARC-780 are the ligands that led to the discovery of the αD pocket^11^ and of the N-terminal segment site^18^ as illustrated in part c of this figure. (c) Overlay of several CK2α and CK2α′ structures in complex with various ligands in order to illustrate the known binding sites of the two isoenzymes. The structure pictures of the figure were prepared with PyMol, version 1.7.^23^

Protein kinase CK2 (acronym derived from the previous name “casein kinase 2”)‡‡Non-standard abbreviations and acronyms: CK2, casein kinase 2; CK2α, catalytic subunit of CK2; CK2α′, paralogous isoform of human CK2α; CK2β, regulatory subunit of CK2; *CSNK2A1*, gene of human CK2α; *CSNK2A2*, gene of human CK2α′; *CSNK2B*, gene of human CK2β; FRET, Förster resonance energy transfer; PDB, protein data bank., an acidophilic Ser/Thr kinase belonging to the CMGC family of eukaryotic protein kinases (EPKs)^1^ and to the minimal kinome essential for all eukaryotic cells,^2^ occurs in *Homo sapiens* predominantly as a heterotetrameric holoenzyme,^3^ in which two catalytic subunits are docked to a homodimer of regulatory subunits (Fig. 1a). The human genome contains two paralogous genes – *CSNK2A1* and *CSNK2A2* – for catalytic CK2 chains, whose products are referred to as CK2α and CK2α′, but only one gene (*CSNK2B*) for the regulatory subunit called CK2β. The sequences of human CK2α and CK2α′ are about 82% identical in the first 330 residues while the two C-terminal segments differ largely in length and sequence. Knockout and other studies have shown that the two isoenzymes cannot fully complement each other.^4^ The central CK2β dimer of the CK2α_2_β_2_ holoenzyme is a permanent and obligatory protein complex based on a zinc-finger stabilized dimerization interface (Fig. 1a). The protein–protein interactions of this CK2β dimer with CK2α or CK2α′, however, are less strong and non-obligatory: CK2α or CK2α′ in a CK2β-unbound state are catalytically active as well, albeit with altered substrate specificity.^5^

Several tumours and a growing number of other diseases are associated with CK2, mostly with overexpression of the enzyme, but increasingly also with dysfunction or deficiency due to genetic defects.^6^ Accordingly, there are many different strategies for efficiently and selectively inhibiting CK2 using small molecules.^7^ For this purpose, a number of ligand binding sites at CK2α/CK2α′ are available (Fig. 1c) apart from the canonical ATP/GTP cavity (unlike most EPKs, in case of CK2, GTP is also a suitable cosubstrate for the kinase reaction in addition to ATP^8^). The best-known ATP-competitive CK2 inhibitors are CX-4945^9^ and SGC-CK2-1^10^ (Fig. 1b), which are also used as control and benchmark inhibitors in many CK2 studies. Not far from the ATP/GTP cavity, the αD pocket is located (Fig. 1c),^11^ a distinctive feature of CK2α/CK2α′, as revealed by a comparative survey of allosteric small-molecule binding sites in EPKs.^12^ Several bivalent CK2 inhibitors have shown that addressing the αD pocket in parallel can improve the affinity and selectivity of ATP-competitive CK2 inhibitors.^13–17^ Three further sites indicated in Fig. 1c – the N-terminal segment site^18^ and the two binding sites of the substrate-competitive CK2 inhibitor heparin^19–21^ – are known so far from crystallographic studies only; whether and how these regions can be exploited for inhibitor design is unexplored.

In the context of efforts to manipulate CK2 function *via* small molecule ligands, the CK2β interface of CK2α/CK2α′ has played a noticeable, but subordinate role so far. Although various peptidic and non-peptidic substances have been presented that interfere with the CK2α/CK2β interaction,^24–33^ none of these compounds has yet come close to being used or even clinically tested. Noteworthy, the CK2β binding sites of CK2α and CK2α′ differ significantly in their affinities to CK2β and smaller ligands,^34^ suggesting that this region might have the potential to enable selective manipulation of the two isoenzymes.

A pioneering CK2β-antagonistic compound was the cyclic peptide Pc (Fig. 1b and c).^25^ Pc was designed on the basis of the CK2α_2_β_2_ holoenzyme structure (Fig. 1a)^22^ from CK2β's interface region to CK2α and showed the expected effect on the CK2α/CK2β interaction in several assays.^25^ Iodination of Pc at its critical hot-spot residue Phe190 – leading to I-Pc (Fig. 1b) – further increased its CK2β-mimicking efficacy.^27^ For later cellular studies, Pc and I-Pc were coupled to cell-penetrating peptides, which resulted in cytotoxic effects in tumour cell lines.^27,28,32,33^

The work described here originates from a crystal structure of CK2α in complex with Pc,^35^ which enabled the development of a fluorescence anisotropy (FA)-based displacement assay optimized for high-throughput screening (HTS).^29^ We present here the application of this assay for the first HTS campaign to discover CK2β-antagonists in an extended compound library (more than 67 000 compounds), the validation of the most promising hits using novel, specially developed FRET (Förster resonance energy transfer) assays, and finally the rationalization of their functionality by complex structures with CK2α and CK2α′.

## Results and discussion

### HTS of a compound library to identify inhibitors of the CK2α/CK2β interaction

**Fig. 2:**
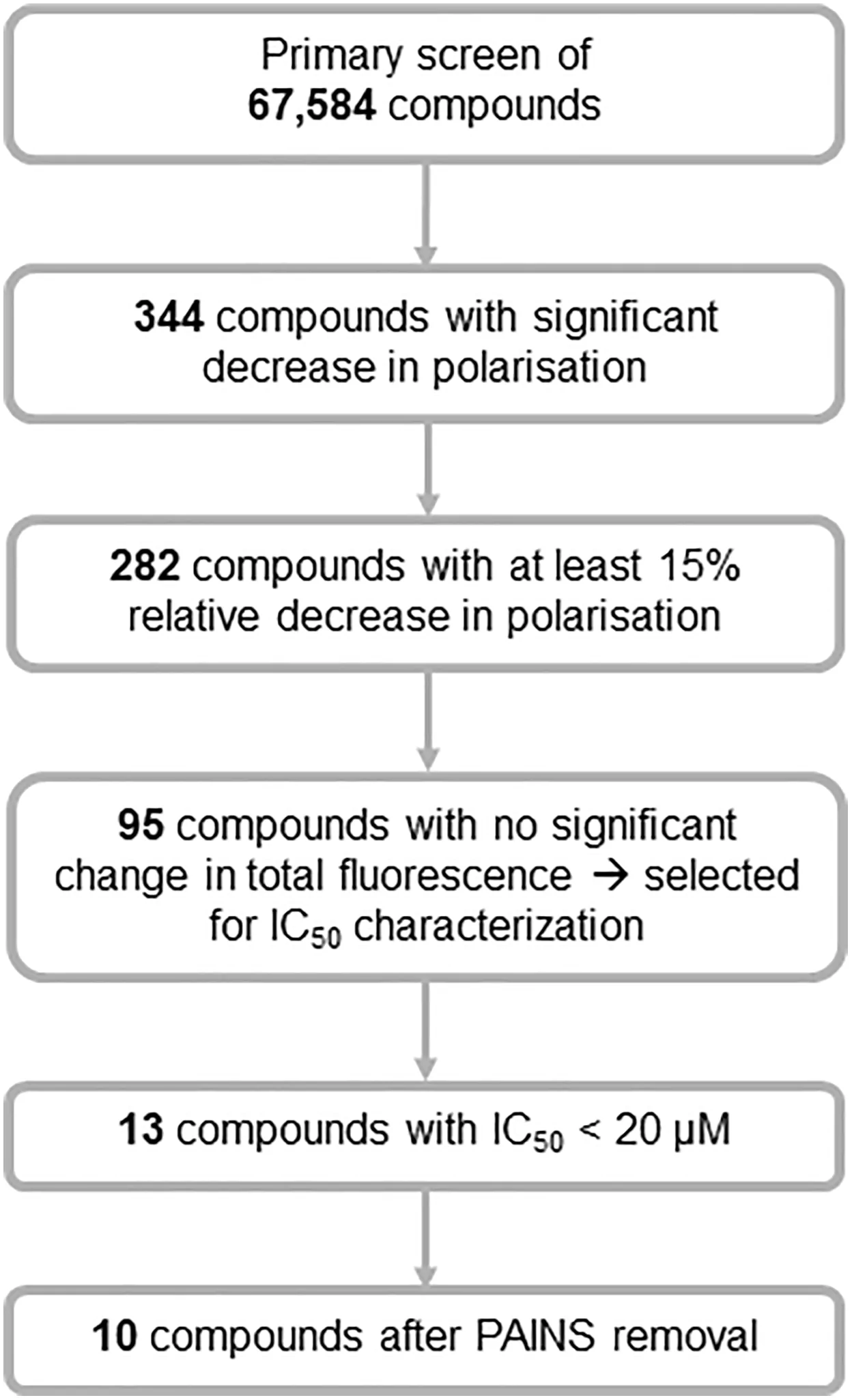
Primary screen of 67 584 compounds for displacement of CF-Ahx-Pc from CK2α^1–335^ using the fluorescence anisotropy assay (*Z*′ = 0.64 ± 0.10, mean value ± SD, *n* = 198).

The FA-based CK2 assay mentioned above^29^ was used to screen a library of 67 584 compounds (FMP Berlin, Germany) for their ability to displace 5,6-carboxyfluorescein-labelled Pc, CF-Ahx-Pc, from CK2α^1–335^ in order to identify novel CK2β-antagonistic compounds (Fig. S1). Displacement of CF-Ahx-Pc from CK2α^1–335^ resulted in a decreased polarization or anisotropy, with 95 compounds of the library showing a significant relative decrease in polarisation of at least 15% but no significant change in total fluorescence and, therefore, were selected for further IC_50_ characterisation (Fig. 2). Dose–response experiments revealed 13 compounds with IC_50_ values of less than 20 µM, with three of them being discarded due to PAINS alerts.^36^ Nine of the remaining ten hits (*i.e.* compounds 2, 4, 5, 8, 12, 14, 15, 18 and 20, Tables 3 and S1) contain the same cyclic pentapeptidic scaffold ((*S*)-Ala-(*S*)-βPhe-(*S*)-Met-(*S*)-Val-4-(*S*)-amino-Pro), differing only in the substituent at the amino group of the 4-aminoproline. The I-Pc^27^ (Fig. 1b) served as a reference competitor exhibiting an IC_50_ value of 0.885 µM and a *K*_i_ value of 0.0669 µM in the presence of 1 µM CK2α^1–335^. When the CK2α^1–335^ concentration was raised to 3 µM (as previously used for competitor characterisation), a *K*_*i*_ value of 0.191 µM was obtained. Both *K*_*i*_ values were found to be not significantly different from the value of 0.158 µM reported by Lindenblatt *et al.*^27^ (Table S1).

### Development of a FRET assay to probe the CK2α/CK2β interaction

The HTS was based on the hypothesis that compounds able to displace the fluorescent peptide CF-Ahx-Pc from the CK2β interface of CK2α would presumably also exert a competitive effect against the entire CK2β protein. In order to rigorously test this for the HTS hits, it was necessary to measure their impact on the CK2α/CK2β and the CK2α′/CK2β interactions quantitatively. In previous studies, we had applied isothermal titration calorimetry (ITC) to quantify the CK2α/CK2β or the CK2α′/CK2β affinity^34,37,38^ and a competitive ITC approach had also served to determine the *K*_*i*_ value of Pc for the CK2α/CK2β interaction.^35^ ITC offers significant advantages (no labelling, no surface mounting, no additives to suppress surface artifacts), but it was not an option here due to its substantial material requirements. This issue particularly affected the key hit compounds of the HTS, which were only available commercially and in limited quantities.

**Fig. 3:**
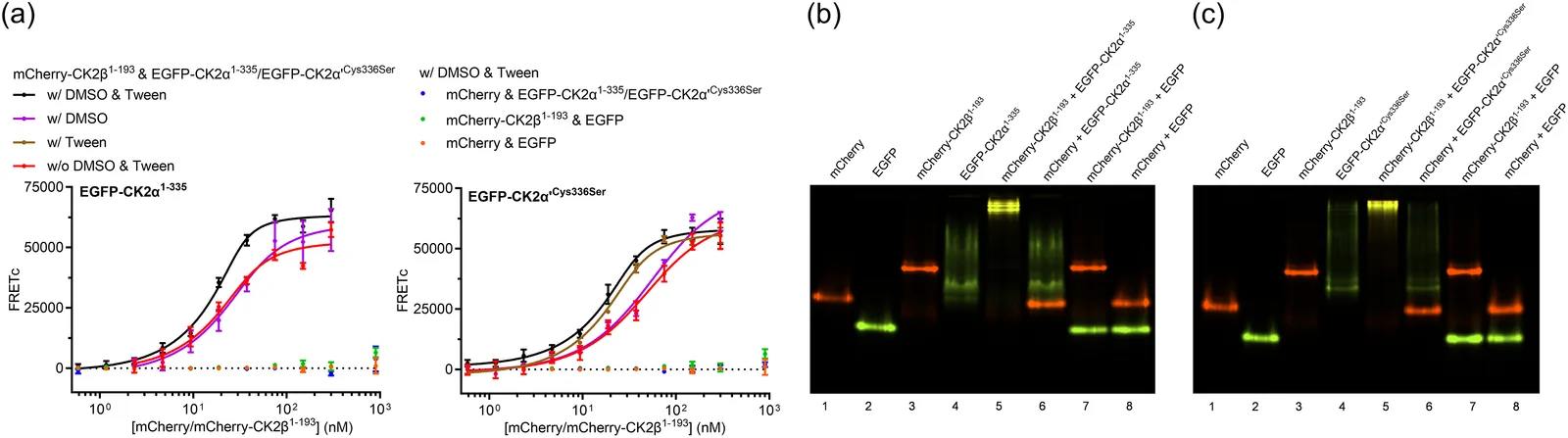
Binding of mCherry-CK2β^1–193^ to EGFP-CK2α^1–335^ ((a), left; (b)) or EGFP-CK2α′^Cys336Ser^ ((a), right; (c)) investigated by FRET measurements on a Synergy™ 2 plate reader (BioTek, USA) (a) and native PAGE (b) and (c), respectively. (a) Mean values ± SEM of three to four experiments each performed in triplicate were obtained without and with 2% (v/v) of DMSO and 0.05% (v/v) of Tween 20 as well as in the presence of one of these two cosolvents. The calculated *K*_D_ values are shown in Table 1. Control experiments were performed to rule out unwanted protein–protein interactions, using either one of the labelled CK2 subunits and EGFP or mCherry, or the two fluorescent proteins alone. Coefficients of determination for all curve fits are provided in Table S4. (b) and (c) Fluorescence of the native PAGE gels was analysed with an Invitrogen™ iBright™ FL1500 imaging system. Shown is an overlay of the green (EGFP and EGFP-labelled proteins) and red (mCherry and mCherry-labelled proteins) channels, with CK2 subunit interaction resulting in yellow bands (lane 5 in (b) and (c), respectively).

Therefore, a homogeneous FRET assay was developed from scratch in two variants to analyse the interaction of either CK2α or CK2α′ with CK2β as well as the inhibition of these protein–protein interactions in both a 96- and 384-well microplate format. For this purpose, the subunits CK2α^1–335^ (CK2α′^Cys336Ser^) and CK2β^1–193^ were genetically fused to EGFP and mCherry, respectively, and heterogeneously expressed in *E. coli* BL21(DE3). Binding of the subunits was characterised both quantitatively by calculating the corrected FRET signal (FRETc) and qualitatively by native PAGE (Fig. 3). The *K*_D_ value of 2.8–8.8 nM (Table 1) for binding of mCherry-CK2β^1–193^ to EGFP-CK2α^1–335^ is in accordance with reported values obtained by ITC (3.7 nM)^37^ and SPR (5.4 nM),^39^ with both co-solvents showing no influence on the dissociation constant. Furthermore, we obtained a *K*_D_ value of 36 nM for the EGFP-CK2α′^Cys336Ser^/mCherry-CK2β^1–193^ interaction, confirming the dissociation constant of 34 nM determined recently by ITC.^34^ However, the latter protein–protein interaction was affected by the presence of 0.05% (v/v) Tween 20 (although not significantly), increasing the proteins’ affinity by an order of magnitude (*P* = 0.0643), whereas 2% (v/v) DMSO showed no impact. Since Tween 20 and DMSO are necessary to maintain a stable signal in the 384-well microplate format and guarantee the solubility of investigated ligands, respectively, all further FRET experiments were conducted in the presence of the two co-solvents. The 5-fold higher affinity of CK2β^1–193^ to CK2α^1–335^ in comparison to that to CK2α′^Cys336Ser^ observed in the FRET experiments without addition of co-solvents (6.7 *vs.* 36 nM) was reflected by the binding behaviour of CK2β-derived peptide CF-Ahx-Pc to the two catalytic CK2 subunits, showing an 8-fold lower *K*_D_ value on CK2α^1–335^ (0.66 *vs.* 5.3 µM, Fig. S2a) in the FA assay.

**Table 1:** Dissociation constants for binding of mCherry-CK2β^1–193^ to EGFP-CK2α^1–335^ and EGFP-CK2α′^Cys336Ser^, respectively

Conditions	EGFP-CK2α^1–335^	EGFP-CK2α′^Cys336Ser^
	*K*_D_ (nM)^a^	*K*_D_ (nM)^b^
without DMSO & Tween 20	6.7 ± 1.7^c^	36 ± 11^d^
with 2% (v/v) DMSO	8.8 ± 3.0	42 ± 9
with 0.05% (v/v) Tween 20	n.d.	5.7 ± 2.0
with DMSO & Tween 20	2.8 ± 1.4	4.2 ± 1.3

a Reported dissociation constants (mean values ± SEM, *n* = 3–4) for binding of CK2β^1–193^ to CK2α^1–335^ in the absence of DMSO and Tween 20 were 3.7 nM (ITC)^37^ and 5.4 nM (SPR).^39^

b Reported dissociation constants (mean values ± SEM, *n* = 3–4) for binding of CK2β^1–193^ to CK2α′^Cys336Ser^ in the absence of DMSO and Tween 20 was 34 nM (ITC).^34^

c *K*_D_ values obtained with DMSO and with DMSO & Tween 20 were not significantly different from the respective values without DMSO & Tween 20 as analyzed by unpaired one-way ANOVAs with Dunnett's multiple comparisons test (adjusted *P* values of 0.7558 and 0.3924, respectively).

d *K*_D_ values obtained with DMSO, with Tween 20 and with DMSO & Tween 20 were not significantly different from the respective values without DMSO & Tween 20 as analyzed by unpaired one-way ANOVAs with Dunnett's multiple comparisons test (adjusted *P* values of 0.9246, 0.0643, and 0.0525, respectively).

To exclude unwanted protein–protein interactions in the FRET experiments either between the fluorescent proteins themselves or between them and the CK2 subunits, different combinations of fluorescent proteins and subunits were analysed for an increase in the FRET signal (Fig. 3a). None such unwanted interactions were detected up to a concentration of 300 nM mCherry or mCherry-CK2β^1–193^, with 1000 nM of these two proteins resulting in a minimal signal increase. Specific CK2 subunit interaction (ratio ∼1 : 1) was confirmed by native PAGE for both EGFP-CK2α^1–335^/mCherry-CK2β^1–193^ (Fig. 3b and Fig. S3a) and CK2α′^Cys336Ser^/mCherry-CK2β^1–193^ (Fig. 3c and Fig. S3b), showing the protein complexes as slow-migrating bands with overlaid green and red fluorescence (lane 5 in Fig. 3b and c each), and by the FRET assay for EGFP-CK2α^1–335^/mCherry-CK2β^1–193^ (Fig. S4), respectively.

**Fig. 4:**
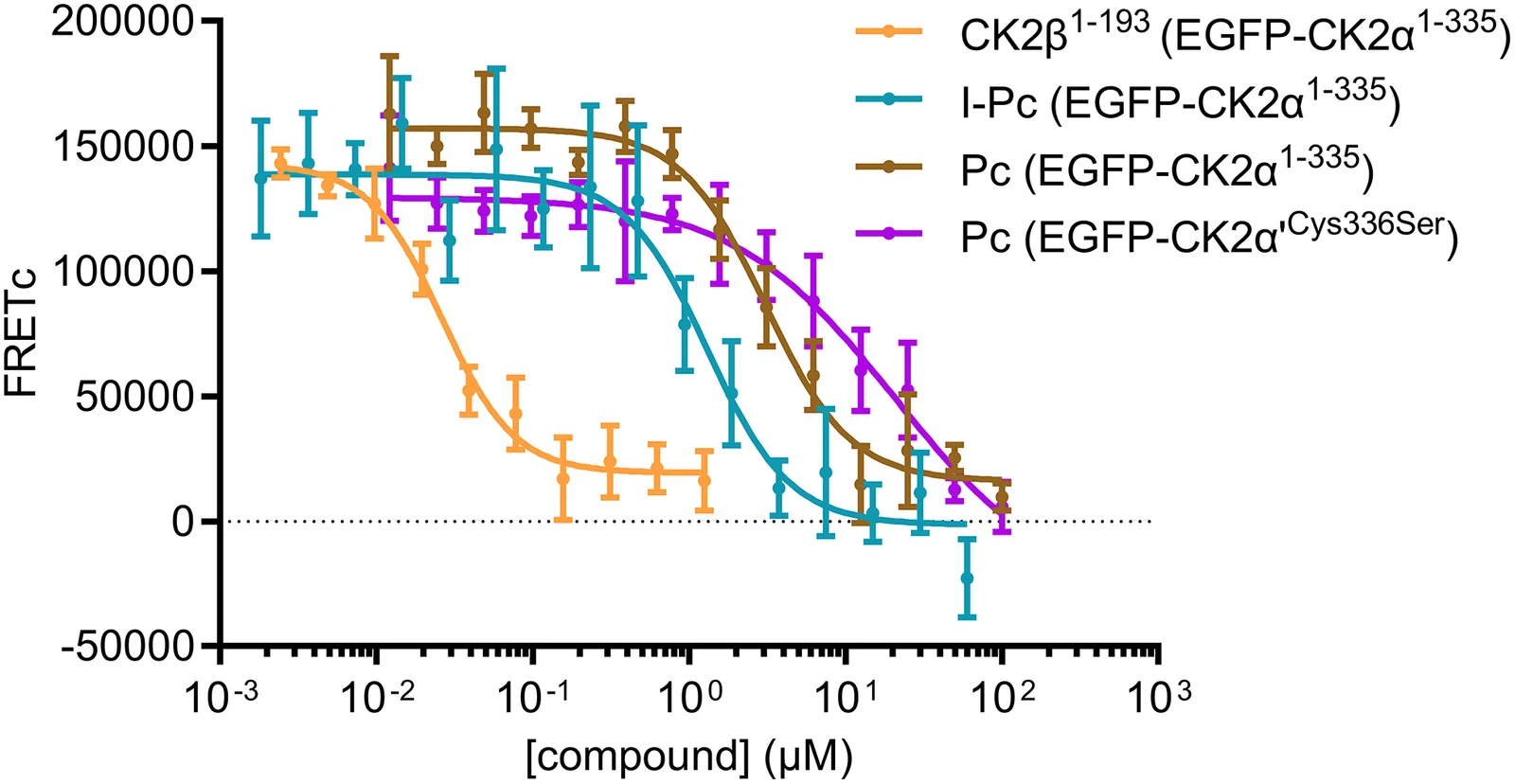
Inhibition of binding of mCherry-CK2β^1–193^ to EGFP-CK2α^1–335^ or EGFP-CK2α′^Cys336Ser^ by reported CK2α/CK2α′ ligands investigated on a Synergy™ 2 plate reader (BioTek, USA). Mean values ± SEM of three experiments, each performed in duplicate or triplicate, are shown. Analysis by the four-parameter equation resulted in IC_50_ values of 0.0255 ± 0.0021 µM (CK2β^1–193^), 1.62 ± 0.53 µM (I-Pc), 3.22 ± 1.03 µM (Pc on EGFP-CK2α^1–335^) and 19.3 ± 6.0 µM (Pc on EGFP-CK2α′^Cys336Ser^). Coefficients of determination for all curve fits are provided in Table S4. Conversion to *K*_*i*_ values reported in Table 2 was done with the “*K*_*i*_ calculator” (https://www.umich.edu/shaomengwanglab/software/calc_ki/index.html) using *K*_D_ values for binding of mCherry-CK2β^1–193^ to two different protein preparations of EGFP-CK2α^1–335^: 6.74 nM (CK2β^1–193^ and I-Pc), 3.28 nM (Pc) and one preparation of EGFP-CK2α′^Cys336Ser^: 7.90 nM (Pc), respectively.

To validate the two FRET assays for the characterization of CK2 subunit interaction inhibitors, we determined the affinity (expressed as *K*_*i*_ value) of known binders to the CK2β site of CK2α^1–335^, *i.e.*, unlabeled CK2β^1–193^,^37,39^ and the cyclic peptides Pc^29,35^ and I-Pc^27,29^ (Fig. 4, Fig. S5a and Table 2). The calculated *K*_*i*_ values of these ligands on EGFP-CK2α^1–335^ were in agreement with previous reports on CK2α^1–335^ (see below). At the same time, the dissociation constant of Pc on EGFP-CK2α′^Cys336Ser^ was confirmed by the respective *K*_*i*_ value on CK2α′^Cys336Ser^ obtained in-house by the FA assay (Fig. S2b). We also studied the interaction of both His-CK2β^1–193^ and Strep-CK2β^1–193^ with EGFP-CK2α^1–335^ (Fig. S5a), showing no influence of either tag on CK2β^1–193^'s binding behavior. Finally, we investigated known ligands of both the ATP binding site (CX-4945^9^ and SGC-CK2-1;^10^Fig. 1b) and the substrate binding site (RRRDDDSDDD^40^ and heparin;^21^Fig. 1c) by the FRET assay, showing no impact of the compounds on the interaction of EGFP-CK2α^1–335^ with mCherry-CK2β^1–193^ (Fig. S5b).

**Table 2:** Reference inhibitors, disturbing binding of mCherry-CK2β^1–193^ to EGFP-CK2α^1–335^ and EGFP-CK2α′^Cys336Ser^, respectively

	EGFP-CK2α^1–335^	EGFP-CK2α′^Cys336Ser^
Compd	*K*_*i*_ (µM)^a^^b^	*K*_*i*_ (µM)^a^^c^
CK2β^1–193^	0.0013 ± 0.0003	n.d.
Pc	0.24 ± 0.08	2.9 ± 0.9
I-Pc	0.21 ± 0.07	n.d.

a *K*_*i*_ values shown are mean values ± SEM, *n* = 3.

b Reported dissociation constants on CK2α^1–335^ were as follows: CK2β^1–193^, 0.0037 µM ITC,^37^ 0.0054 µM (SPR);^39^ Pc, 1.7 µM (FA, Fig. S2b), 0.64 µM (FA),^29^ 0.56 µM (ITC);^35^ I-Pc, 0.16 µM (FA),^27^ 0.24 µM (ITC).^29^

c Reported dissociation constant on CK2α′^Cys336Ser^ was Pc, 3.6 µM (FA, Fig. S2b).

### Validation of the HTS hits

**Fig. 5:**
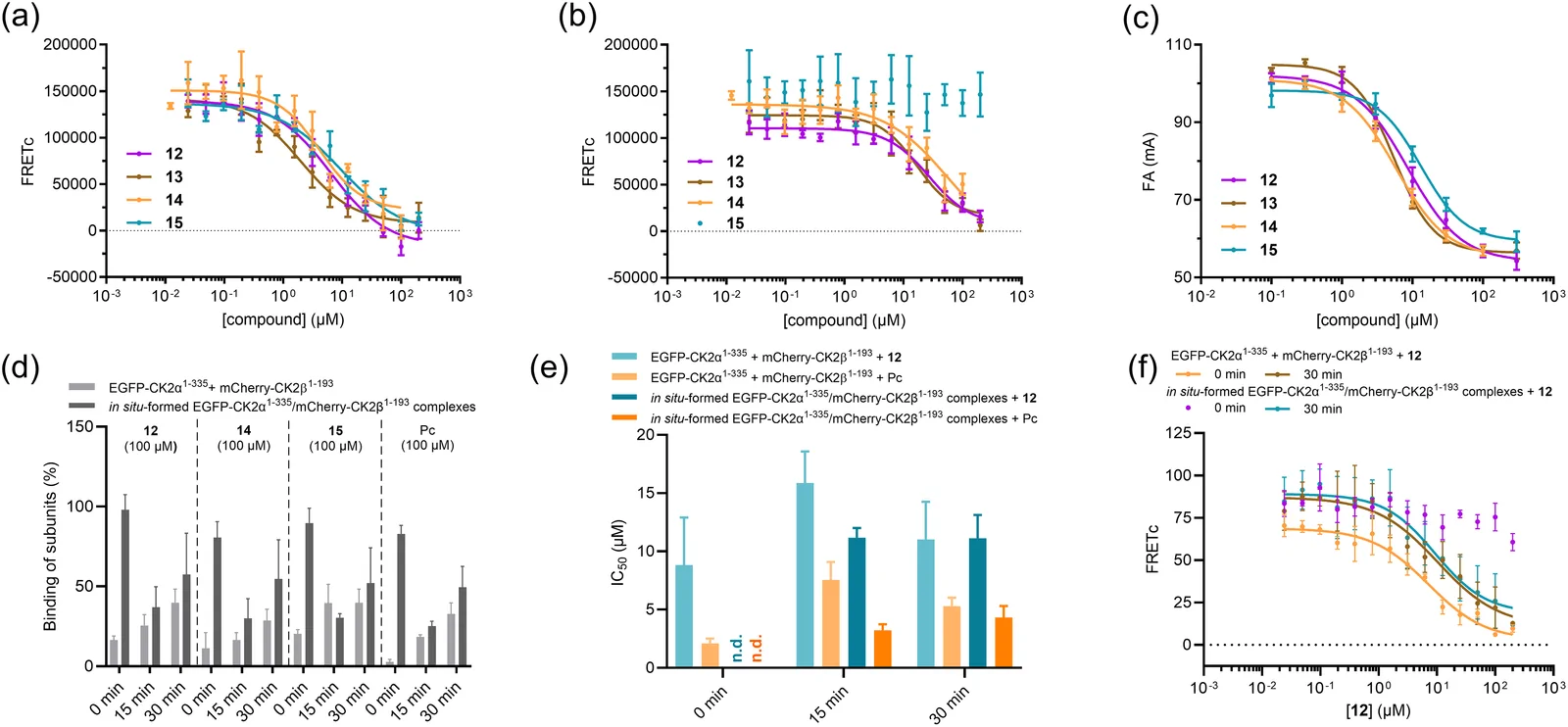
Characterization of cyclic pentapeptides 12–15 and of Pc, as inhibitors of the CK2 subunit interaction. (a) and (b) FRET experiments following binding of mCherry-CK2β^1–193^ to either EGFP-CK2α^1–335^ (a) or EGFP-CK2α′^Cys336Ser^ (b) and FA experiments showing binding of CF-Ahx-Pc to CK2α^1–335^ (c) gave values of IC_50_ and *K*_*i*_ listed in Table S2 and Table 3, respectively. Experiments in (a)–(c) were performed on a Synergy™ 2 plate reader (BioTek, USA). (d) Compounds 12, 14, 15, and Pc (100 µM each) inhibit the formation of EGFP-CK2α^1–335^/mCherry-CK2β^1–193^ complexes (30 nM of each protein) (light grey bars) and break up preformed complexes (30 nM) (dark grey bars). Either the individual CK2 subunits or a pre-incubated mixture of CK2 subunits (30 min at 30 °C) was added to the compounds, and FRET was investigated either directly after addition of the proteins or after 15 min and 30 min of incubation, respectively. (e) IC_50_ values for either the inhibition of binding of mCherry-CK2β^1–193^ to EGFP-CK2α^1–335^ using the individual CK2 subunits (light bars) or the disruption of *in situ*-formed EGFP-CK2α^1–335^/mCherry-CK2β^1–193^ complexes (dark bars) by compound 12 (cyan) and Pc (orange). FRET was investigated immediately after the addition of either the CK2 subunits or a pre-incubated mixture of the CK2 subunits or after 15 min and 30 min of incubation, respectively. A paired one-way ANOVA with Tukey's multiple comparisons test showed non-significant differences (*P* ≥ 0.05) of IC_50_ values for either of the two inhibitors after 0, 15, and 30 min using the individual CK2 subunits and after 15 and 30 min using the pre-formed CK2 complex, respectively. An unpaired one-way ANOVA with Tukey's multiple comparisons test of the IC_50_ values obtained with the individual CK2 subunits and the CK2 complex after either 15 or 30 min of incubation showed a significant difference only for Pc after 15 min of incubation (*P* = 0.0489), while all the other comparisons resulted in non-significant differences (*P* ≥ 0.05). (f) Dose response curves for either the inhibition of the formation of EGFP-CK2α^1–335^/mCherry-CK2β^1–193^ complexes or for the breakup of pre-formed complexes. Measurements were done without incubation and with 30 min of pre-incubation of proteins and compound 12, respectively. IC_50_ values of 12 were as follows: individual CK2 subunits: 8.83 ± 4.08 µM (0 min), 15.9 ± 2.7 µM (15 min, not shown), 11.0 ± 3.2 µM (30 min); CK2 complex: 11.2 ± 0.8 µM (15 min, not shown), 11.1 ± 2.0 µM (30 min). Experiments in (d)–(f) were performed on an Infinite® M1000 PRO plate reader (Tecan Group, Switzerland). Coefficients of determination for all curve fits are provided in Table S4.

The initial screening had provided a new cyclic pentapeptidic scaffold ((*S*)-Ala-(*S*)-βPhe-(*S*)-Met-(*S*)-Val-4-(*S*)-amino-Pro) able to bind to the CK2β site of CK2α^1–335^, with compound 12 carrying a 2-chloro-5-methylbenzoyl substituent at the 4-(*S*)-aminoproline being the most potent binder (Table S1). The initially discovered 9 cyclic pentapeptides together with another 19 derivatives varying in both the amino acid composition of the cyclic part and the substituent at the amino group of the 4-(*S*)-aminoproline (commercially obtained from ChemBridge Corporation, San Diego, CA, USA) were further investigated for binding on CK2α (FA assay) as well as for inhibition of the CK2α/CK2β and CK2α′/CK2β interactions (FRET assays). Results are provided in Table 3, Table S2 and Fig. 5a–c.

**Table 3:** Characterization of cyclic pentapeptides as ligands of the CK2β site of CK2α^1–335^, preventing binding of mCherry-CK2β^1–193^ to both EGFP-CK2α^1–335^ and EGFP-CK2α′^Cys336Ser^

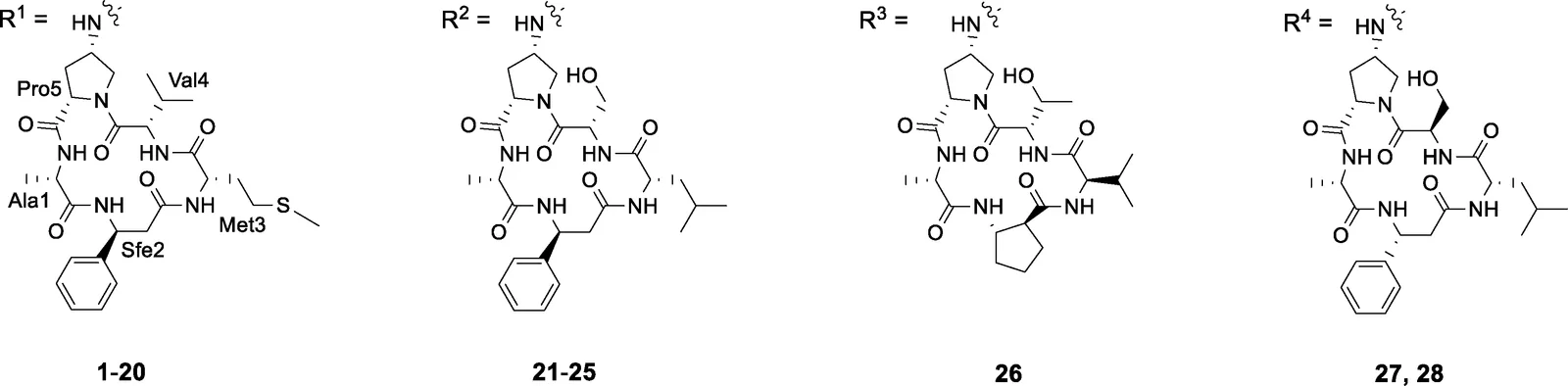				
Compd	Structure	CK2α^1–335^	EGFP-CK2α^1–335^	EGFP-CK2α′^Cys336Ser^
		*K*_*i*_ (µM)^a^	*K*_*i*_ (µM)^a^	*K*_*i*_ (µM)^a^
1	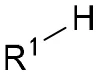	>70^b^	∼15	>30^c^
2	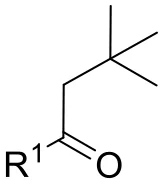	8.0 ± 0.8	2.5 ± 0.1	>15^d^
3	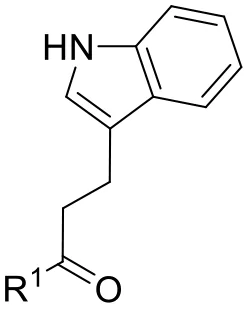	>23^d^	∼7.5	>15^d^
4	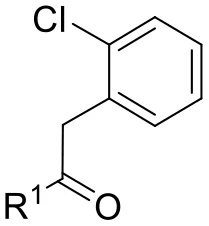	21 ± 3	3.2 ± 0.9	>3.0^e^
5	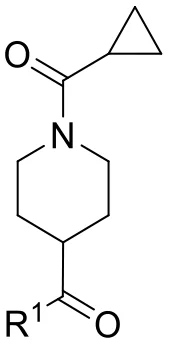	11 ± 2	1.6 ± 0.4	14 ± 2
6	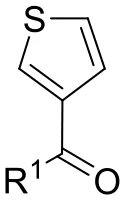	9.8 ± 0.1	2.5 ± 0.8	16 ± 3
7	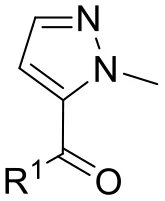	14 ± 2	7.7 ± 0.7	>3.0^e^
8	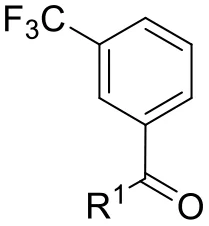	4.9 ± 0.6	1.2 ± 0.1	8.7 ± 1.4
9	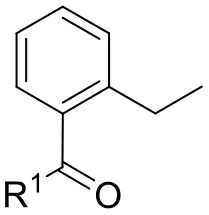	2.2 ± 0.4	0.80 ± 0.21	6.1 ± 1.4
10	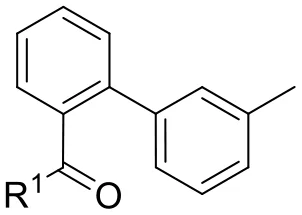	2.5 ± 0.1	0.59 ± 0.18	>3.0^e^
11	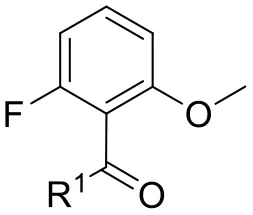	2.2 ± 0.2	0.49 ± 0.03	5.1 ± 1.1
12^f^	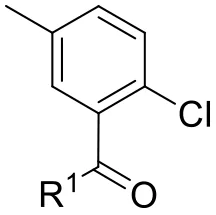	2.0 ± 0.5	0.57 ± 0.16	4.5 ± 0.9
13	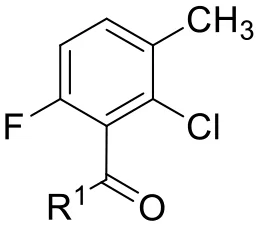	0.92 ± 0.12	0.15 ± 0.01	2.4 ± 0.3
14	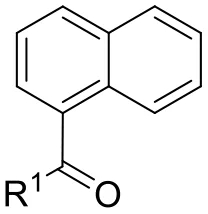	0.99 ± 0.23	0.32 ± 0.07	8.5 ± 0.8
15^f^	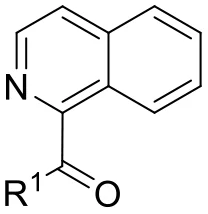	2.7 ± 0.4	0.74 ± 0.09	>30^c^
16	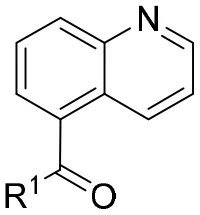	2.4 ± 0.4	1.0 ± 0.3	11 ± 3
17	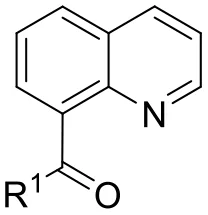	9.3 ± 2.3	1.5 ± 0.6	>30^c^
18	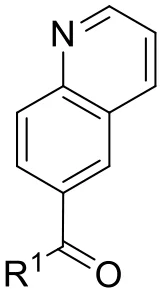	∼23	2.1 ± 0.3	>15^d^
19	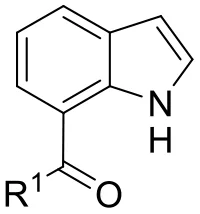	5.1 ± 0.1	1.7 ± 0.2	>15^d^
20	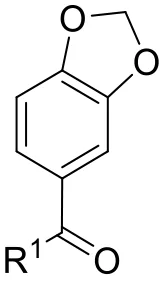	7.3 ± 2.7	2.3 ± 0.2	17 ± 4
21	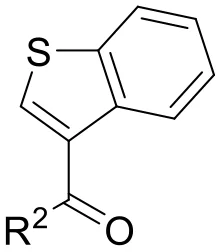	>23^d^	>7.5^d^	>15^d^
22	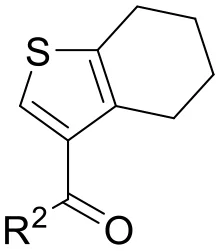	>23^d^	>7.5^d^	>15^d^
23	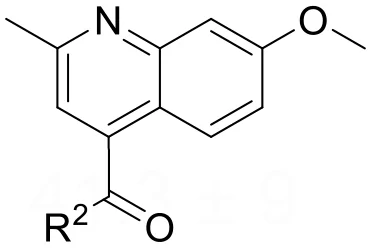	>23^d^	>7.5^d^	>15^d^
24	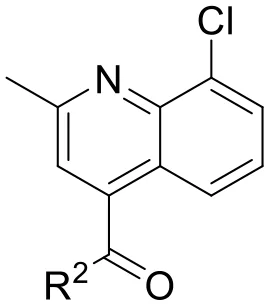	>70^b^	>15^c^	>15^d^
25	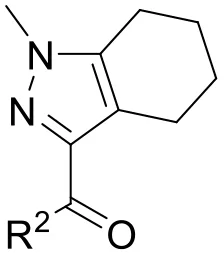	>70^b^	>15^c^	>15^d^
26	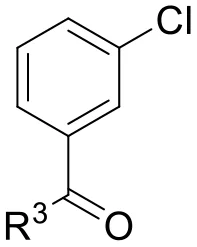	>70^b^	>15^c^	>15^d^
27	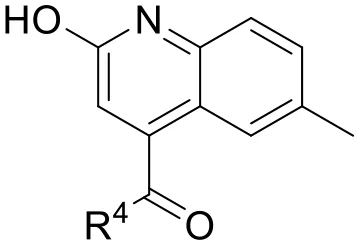	>70^b^	>15^c^	>15^d^
28	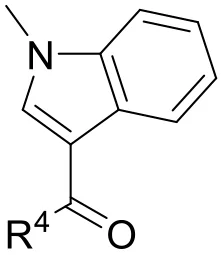	>70^b^	>15^c^	>15^d^

a Dissociation constants *K*_*i*_ (mean values ± SEM, *n* = 3–4) were calculated from the respective IC_50_ values (see Table S2).

b Highest compound concentration investigated yielding less than 50% inhibition was 300 µM.

c Highest compound concentration investigated yielding less than 50% inhibition was 200 µM^c^.

d Highest compound concentration investigated yielding less than 50% inhibition was 100 µM.

e Highest compound concentration investigated yielding less than 50% inhibition was 20 µM.

f Compounds 12 and 15 were co-crystallized with CK2α^1–335^ and CK2α′^Cys336Ser^. In the PDB (consortium, 2019), the two compounds are defined as cyclic peptides with “SFE” used as three-letter code for (*S*)-β-phenylalanine. In the novel 5-character coding system, which the PDB introduced for new ligands, the derivatised proline residues at position 5 received the codes A1ICB (**12**) and A1ICC (**15**), respectively.

Exchange of amino acids within the identified cyclic pentapeptide (present in compounds 1–20) as in compounds 21–25 (exchange of (*S*)-Met and (*S*)-Val for (*S*)-Leu and (*S*)-Ser), 26 (exchange of (*S*)-βPhe = Sfe, (*S*)-Met and (*S*)-Val for 2-(*S*)-amino-cyclopentane-1-(*S*)-carboxylic acid, (*R*)-Val and (*S*)-Thr), and 27 and 28 (exchange of (*S*)-βPhe = Sfe, (*S*)-Met and (*S*)-Val for (*R*)-βPhe, (*S*)-Leu and (*R*)-Ser) led to complete loss of binding activity on CK2 subunits. Furthermore, substitution of the 4-(*S*)-aminoproline was found to be crucial for activity (the unsubstituted derivative 1 is inactive), which is why we focused on compounds 2–20. In general, these cyclic pentapeptides showed higher affinity (*i.e.*, a lower *K*_*i*_ value) for disturbing the CK2α/CK2β interaction compared to the CK2α′/CK2β interaction. Considering the 10 compounds 5, 6, 8, 9, 11–14, 16, and 20, for which exact *K*_*i*_ could be calculated for inhibition of the two protein–protein interactions, the difference in affinity was 6.4- to 27-fold. This was only exceeded by derivative **15** substituted with an isoquinoline-1-carboxylic moiety at the 4-(*S*)-aminoproline, which exhibits more than 40-fold selectivity for CK2α over CK2α′ (Table 3, Table S2 and Fig. 5a and b).

Structurally, compounds 2–20 can be divided into three series either carrying aliphatic (2–5), monocyclic (hetero-)aromatic (6–13) or bicyclic (hetero-)aromatic (14–20) carboxylic substituents at the 4-(*S*)-aminoproline. While the second substitution pattern was in general beneficial for both CK2α and CK2α′ binding, with compounds 12 and 13 (Fig. 5a–c) being overall most promising, derivatives with bicyclic (hetero-)aromatic carboxylic substituents, particularly compounds 14 and 15 (Fig. 5a–c), were potent and selective CK2α ligands.

So far, we had investigated the influence of peptides on the formation of CK2α/CK2β or CK2α′/CK2β complexes. However, Pc^25^ and I-Pc^29^ (Fig. 1) (as well as the equipotent I-Pc derivative sc18-I-Pc^27^) are known to also induce dissociation of pre-formed CK2α_2_β_2_ holoenzyme. This ability was previously proven directly by using radioactively labelled CK2α and immobilized CK2β and indirectly by phosphorylation of CK2β-dependent CK2 substrates, respectively. According to an established classification,^5^ CK2 substrates requiring CK2β as part of the enzyme are called “class III” substrates in contrast to “class II” substrates (phosphorylation only with CK2β-free CK2α) and “class I” substrates (phosphorylation with either CK2α or the CK2α_2_β_2_ holoenzyme as a catalyst). Herein, we applied the newly developed FRET assay with EGFP-CK2α^1–335^ and mCherry-CK2β^1–193^ for directly investigating the compounds’ ability to disrupt pre-formed CK2α/CK2β complexes. Based on our native PAGE experiments (Fig. 3b and Fig. S3a), we pre-incubated equimolar concentrations (30 nM) of EGFP-CK2α^1–335^ and mCherry-CK2β^1–193^ for 30 min to guarantee complete complex formation, before a CK2α ligand (Pc, 12, 14 or 15; 100 µM) was added (Fig. 5d). To our surprise, Pc was found to be almost inactive directly after addition to the reaction mixture. Only a second incubation step of 15 or 30 min led to inhibition of CK2 subunit interaction to similar extent as seen in experiments with the separate CK2 subunits. The identical behaviour was observed when studying the cyclic pentapeptides 12, 14 and 15.

To validate the results determined with the FRET assay by an established method, we followed CK2-catalyzed phosphorylation of RRRDDDSDDD (class I substrate according to a classification introduced by Pinna^5^) and eIF2β^1–22^ (class III substrate) by γ-^32^P-ATP as previously reported by Lindenblatt *et al*.^27^ Enzyme was either full-length CK2α in the presence of equimolar full-length CK2β (90 nM each) or CK2α_2_β_2_ holoenzyme (10 nM), with the enzymatic reaction being started directly after mixing enzyme and inhibitor (Fig. S6a) or after 10 min of pre-incubation of enzyme and inhibitor (Fig. S6b) by addition of ATP and the respective substrate peptide. Without pre-incubation, compounds 12, 14, and 15 (100 µM) led to a considerable decrease in phosphorylation of both peptide substrates when CK2α and CK2β were individually added to the reaction mixture. This behaviour reflects the inhibition of the CK2 subunit interaction and, thus, the stimulatory effect of CK2β on CK2α′s kinase activity towards RRRDDDSDDD^40^ and emphasizes the requirement of the regulatory CK2β subunit for CK2α-catalysed eIF2β^1–22^ phosphorylation,^41^ respectively. In contrast, the three cyclic pentapeptides had no impact on the activity of a pre-formed CK2α/CK2β complex, *i.e.*, the holoenzyme, without pre-incubation (Fig. S6a), which confirmed the respective result of the FRET assay (Fig. 5d). When enzyme and inhibitor were pre-incubated for 10 min (Fig. S6b), we obtained, however, comparable results as without the pre-incubation step, which was not in line with the FRET assay (Fig. 5d). This difference might be attributed to the nature of the CK2α/CK2β complex used in the two assays. While this complex was formed *in situ* for the FRET experiments by pre-incubating the CK2 subunits, analogous to the procedure reported by Laudet *et al.*^25^ and Iegre *et al.*,^33^ a purified CK2 holoenzyme was applied in the radiometric assay, which may require longer pre-incubation with CK2 subunit interaction inhibitors to dissociate.

To quantify the potency of CK2α ligands to interfere with both CK2 subunit association and dissociation, we repeated the FRET experiment with Pc and compound 12, respectively, in a dose-dependent manner (Fig. 5e, f and Fig. S7). We confirmed the complete loss of activity for the two CK2α ligands when studying complex dissociation without a second incubation step. Furthermore, both Pc and compound 12 became equipotent regarding their influence on association and dissociation after 30 min of incubation with the individual CK2 subunits and the pre-formed CK2 complex, respectively. This result indicates an equilibrium between the ligands, the CK2 subunits and the CK2 complex established after a period of 30 min. The ligand's potency at equilibrium is well described by the association experiment without a second incubation step, *i.e.*, those conditions used for characterizing the reference ligands (Table 2 and Fig. 4, Fig. S5) and the cyclic pentapeptides (Table 3, Table S2 and Fig. 5a) in this work. The pre-incubation (30 min) of the individual CK2 subunits before addition of Pc or compound 12 in the association experiments did not affect the compounds’ potencies, *i.e.*, their IC_50_ values [Pc: 3.22 ± 1.03 µM (no pre-incubation), 2.08 ± 0.43 µM (30 min of pre-incubation); 12: 7.60 ± 2.06 µM (no pre-incubation), 8.83 ± 4.08 µM (30 min of pre-incubation)].

In conclusion, we were able to experimentally confirm that the key HTS hit compounds interfere with the assembly of the CK2 holoenzymes CK2α_2_β_2_ and 
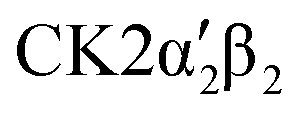
 and also cause their dissociation. It is likely that other protein–protein interactions requiring an intact heterotetrameric CK2 holoenzyme^42^ would also be impaired by these CK2β-antagonistic substances, albeit indirectly; however, this was not investigated experimentally here.

### CK2α and CK2α′ crystal structures in complex with efficient inhibitors of the CK2α/CK2β interaction

To elucidate the binding modes of the hit compounds, we selected compounds 12 and 15 (Table 3 and Table S2) that were among those with the highest affinity and tried to crystallize them together with CK2α^1–335^,^43^ or with CK2α′^Cys336Ser^,^38^*i.e.*, with variants of the two human CK2α paralogs well established for crystallographic purposes.^18^

Three of four possible co-crystal structures – CK2α^1–335^/12, CK2α^1–335^/15, and CK2α′^Cys336Ser^/12 – resulted from these efforts (Table S3). Yet, extensive crystallization attempts were not successful with the pair CK2α′^Cys336Ser^/15 fitting well to the fact that no binding of compound 15 to CK2α′^Cys336Ser^ could be detected with the FRET-assay (Fig. 5b). Among those three complex structures (Table S3), CK2α′^Cys336Ser^/12 has by far the best resolution (1.16 Å): this also matches with established knowledge, namely that complex crystal structures with CK2α′ tend to have significantly better resolutions than their equivalents with CK2α.^18^

**Fig. 6:**
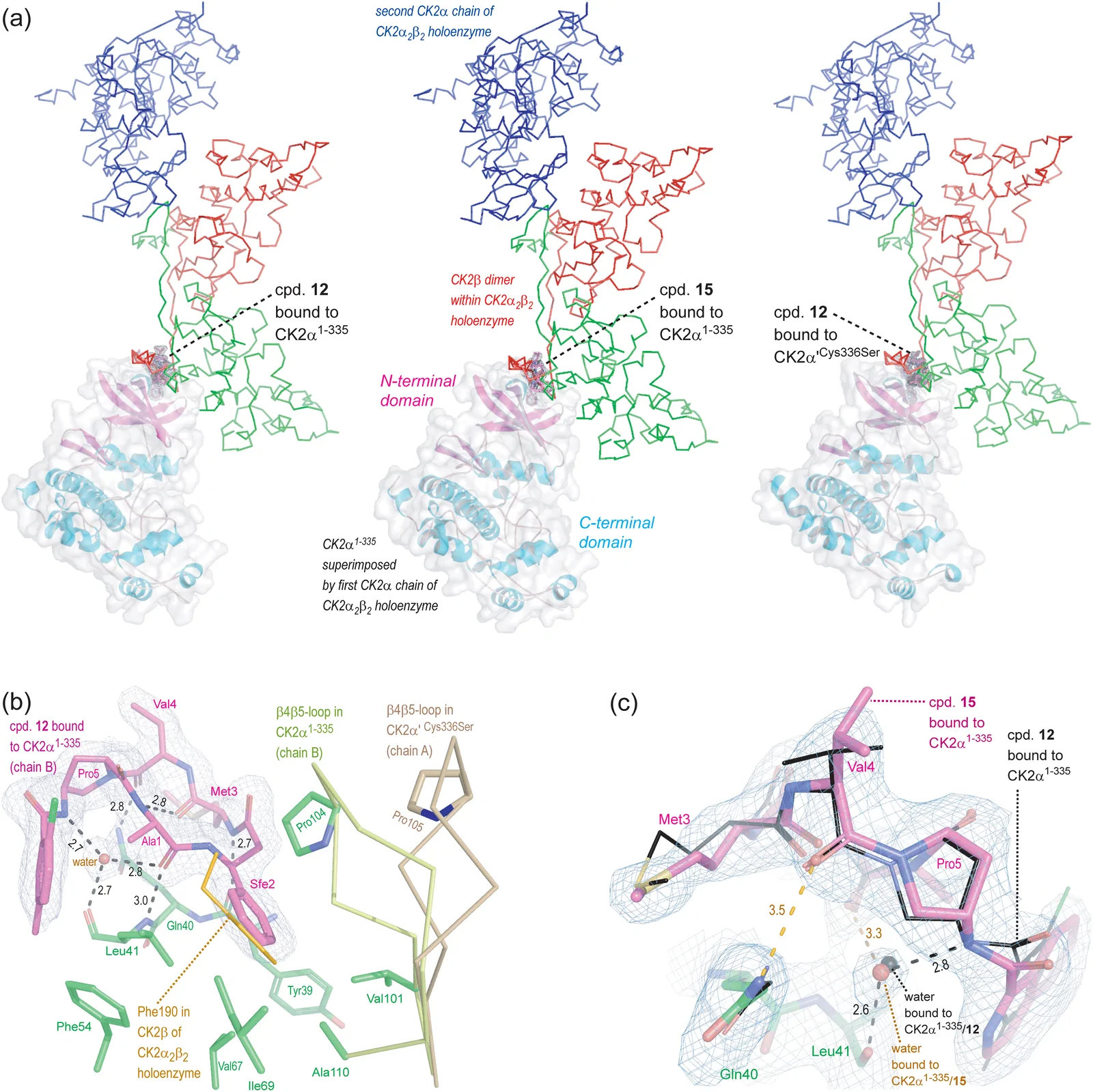
X-ray structures of CK2α^1–335^ and CK2α′^Cys336Ser^ in complex with compounds 12 or 15. (a) Overview of one protomer with bound ligand (covered by electron density with a cutoff level of 1σ) from each of the three complex structures; in each case, the CK2β dimer (red and green traces) and one CK2α chain (blue) from the CK2α_2_β_2_ holoenzyme structure with PDB_ID 1JWH^22^ were depicted after superimposition of the holoenzyme chain A (not drawn for reasons of clarity) on the enzyme part of the complex. (b) Interaction of compound 12 with CK2α (exemplified for one of the three CK2α^1–335^ protomers in the asymmetric unit) in comparison to CK2α′ (from which only the β4β5 loop with Pro105 at its tip was drawn); Phe190 of CK2β was drawn in orange colour after superimposition of chain A of the CK2α_2_β_2_ holoenzyme structure with PDB_ID 4NH1^47^ on the enzyme part of the complex; hydrogen bonds are indicated by black dashed lines (with distances in Å); compound 12 and a hydrogen-bond mediating water molecule are covered by electron density (cutoff level 1σ). (c) The hydrogen-bond mediating water molecule in chain C of the CK2α^1 335^/15 complex (orange) and for comparison in the CK2α^1 335^/12 complex (black); black dashed lines: H-bonds (with distances in Å); orange dashed lines: atomic contacts (with distances in Å) whose equivalents in the CK2α^1 335^/12 complex are H-bonds; the electron density belongs to the CK2α^1–335^/15 complex (cutoff level 1σ). All structure pictures of the figure were prepared with PyMol, version 1.7.^23^

The most important finding from the three complex structures is that they explain the CK2β-competitive nature of compounds 12 and 15: as expected, 12 or 15 bind to the CK2β interface of CK2α or CK2α′, the key feature for the assembly of the CK2α_2_β_2_ holoenzyme located at the N-terminal domain of CK2α or CK2α′ (Fig. 6a). In the two CK2α^1–335^ structures, a peptidic ligand occupies each of the three crystallographically independent protomers (Table S3); in contrast, in the CK2α′^Cys336Ser^ structure, compound 12 is bound only to one of two CK2α′^Cys336Ser^ chains present in the asymmetric unit. On the side of CK2α, Leu41 and Phe54 are the central hotspots of the CK2α/CK2β interaction, as revealed by a combined mutagenesis and ITC study.^37^ The critical role of these two residues is also evident from the complex structures of this work (Table S3), since in these, the hydrophobic stack of Leu41/Phe54 side chains is embraced by compounds 12 (Fig. 6b) and 15 (not shown). This, however, is only possible if the cyclic peptides present a concave surface to the enzyme, for which they must adopt a bent conformation.

This particular conformation in fact exists in all eight crystallographically independent peptides of the three crystal structures of Table S3. It is stabilised by a hydrogen bond across the peptide ring (between Ala1 and Met3) and by a hydrophobic interaction between the methyl group of Ala1 and the aromatic system attached to Pro5, which must be oriented towards each other accordingly (Fig. 6b). In complex with the enzyme, it looks as if this aromatic moiety and the side chain of (*S*)-β-phenylalanine (Sfe2, Fig. 6b) hold the Leu41 side chain like a clamp. Together with further side chains from CK2α′s CK2β interface (Tyr39, Phe54, Val67, Ile69, Val101, Pro104 and Ala110), they form an extended hydrophobic cluster. Its centre is filled by the phenyl ring of Sfe2 which thus adopts the role of the side chain of Phe190 in CK2β: Phe190, the key hot spot of the CK2α/CK2β interaction on the CK2β side,^25^ occupies the equivalent position within the CK2α_2_β_2_ holoenzyme (Fig. 6b).

The importance of the phenyl ring of Sfe2 for binding is emphasised by comparing the affinities (*K*_*i*_ values) of compounds 2 to 20 to CK2α/CK2α′ with those of the largely ineffective substances 27 and 28 (Table 3). Although the interpretation is complicated by the fact that 27/28 also differ from 2–20 in the side chains of the amino acids at positions 3, 4 and 5 (see structural formulas in Table 3), it is nevertheless reasonable to assume that the most important difference is that in 27 and 28, sequence position 2 is occupied by (*R*)-β-phenylalanine instead of (*S*)-β-phenylalanine. The consequence of this chirality change at the C_γ_ atom will be that the phenyl ring, which is rigidly bound to the peptide backbone, points in the wrong direction, so that the pocket in the centre of the hydrophobic cluster at the interface can no longer be adequately filled.

However, the *S*-configuration of β-phenylalanine at ring position 2 may be a necessary condition for a strong CK2β-competitive effect, but it is not a sufficient one. This is obvious from a comparison of compounds 21 to 25 in Table 3 with 2 to 20: compounds 21–25 also have (*S*)-β-phenylalanine at position 2, but overall, they show a significantly weaker effect than compounds 2 to 20. Follow-up studies are required to clarify why this is the case, as 21–25 differ from 2–20 (just like 27/28) at ring positions 3, 4, and 5, leaving no clear candidate. It is merely unlikely that the replacement of Val4 in 2–20 with serine in 21–25 is relevant, because Val4 has no contact with the enzyme in any of the three complex structures elucidated here (Table S3).

### The β4β5 loop is responsible for affinity differences between CK2α and CK2α′ at the CK2β binding site

The above-mentioned fact that Pro104 of CK2α is part of the critical hydrophobic binding cluster at the enzyme/ligand interface is not self-evident, as this residue is located at the tip of the β4β5 loop, which can adopt different conformations in CK2α.^44,45^ The Pro104 side chain touches the hydrophobic cluster only if the β4β5 loop is bent towards the cyclic peptide at the CK2β binding site as visible in Fig. 6b. In contrast, in the CK2α′^Cys336Ser^/12 structure, the β4β5 loop has an open and stretched conformation, in which Pro105 (the equivalent to Pro104 in CK2α) does not contact the ligand (Fig. 6b). At the CK2β binding site, CK2α and CK2α′ have identical amino acid compositions. The conformational deviation of the β4β5 loop is the only clear structural difference in this region between the CK2α^1–335^/12 and CK2α′^Cys336Ser^/12 complexes; therefore, this is most likely the structural basis for the fact that CK2α′ binds compound 12 with about 8-fold lower affinity than CK2α (Table 3 and Table S2) and for the general tendency visible from Table 3 and Table S2 that the affinity of the investigated compounds is significantly lower to CK2α′ compared to CK2α.

Noteworthy, compound 12 is the first ligand at the CK2β binding site at all, for which complex structures are available with both CK2α and CK2α′. This fact, combined with the striking structural difference at the β4β5 loop (Fig. 6b), was the starting point for a recently published study^34^ investigating the reasons for the difference in affinity of the two isoenzymes for ligands at the CK2β interface. A structural survey in that study revealed that the β4β5 loop in CK2α′ always adopts the open conformation, as seen in Fig. 6b. In contrast, in CK2α it can occur in the two principal conformations shown in Fig. 6b. To reduce the structural constraints on the β4β5 loop of CK2α′ and to make it similarly adaptable as in the case of CK2α, a CK2α′ mutant was designed and characterized, in which the back of the β4β5 loop, which has no direct contact with CK2β or CK2β-competitive ligands, was made similar to CK2α. Indeed, this CK2α′ mutant binds CK2β with an affinity similar to that of CK2α as shown *via* ITC,^34^ and the same equivalence was demonstrated using the FA assay^29^ for the Pc peptide.^34^ In summary, these observations support the notion that the β4β5 loop and its structural adaptability have a critical impact on the binding strength of the CK2β interface of CK2α/CK2α′.

Possibly, the adaptability of the β4β5 loop provides also the background for the levelling effect of Tween 20 – the tendency that the CK2β affinity of CK2α′ in the presence of this surfactant approaches that of CK2α as suggested by the data of Table 1. Tween 20 was used here to suppress surface artifacts and thus to enable the broad applicability of the newly developed FRET assays in 384-well microplates. However, it has also been reported that it can have a structural impact on proteins;^46^ therefore, it cannot be excluded that Tween 20 might also increase the adaptability of the β4β5 loop of CK2α′ and thereby indirectly the CK2α′/CK2β affinity.

### A critical water molecule contributes to the enzyme/ligand interaction

The structure of the CK2α^1–335^/12 complex illustrates well that the role of Leu41 is not limited to being part of the hydrophobic binding cluster. Rather, both peptide groups of Leu41 form hydrogen bonds with peptide groups of the ligand (Fig. 6b); they are complemented by two further hydrogen bonds between the enzyme (Tyr39/Gln40) and the ligand (Fig. 6b).

One of these hydrogen bonds of Leu41 is mediated by a water molecule that is well defined by electron density and located at the centre of a small network of hydrogen bonds (Fig. 6b). The key role of this cross-linking water molecule for ligand binding is suggested by two observations: (i) compound 15 has approximately the same affinity to CK2α as compound 12 (Table 3); however, if one compares the CK2α^1–335^/12 and the CK2α^1–335^/15 complex structures in this region (Fig. 6c), it can be seen that among the cross-linking hydrogen bonds, only those mediated by the bound water molecule are retained and apparently essential, whereas for example the H-bond between the side chain of Gln40 and the carbonyl O-atom of Val4 of the ligand, which is well established in the CK2α^1–335^/12 structure (Fig. 6b), is no longer present in the CK2α^1–335^/15 complex (Fig. 6c). (ii) The bound water molecule is shielded from the external solvent by the chemical group that is bound *via* an amide bond to the 4-amino group of Pro5 (columns 2 and 7 of Table 3). Without such a substituent (cpd. 1), there is hardly any CK2β-competitive effect. If the amide bond is followed by a group with a low shielding effect (*e.g.*, a methylene group as in compounds 2/3/4, an aliphatic ring as in cpd. 5, or a small heteroaromatic ring as in compounds 6/7), the efficacy of the ligand is also greatly reduced. However, a ligand is particularly efficient when the substituent is a well-oriented aromatic system of considerable size (extended either by a second ring or by suitable substituents; compounds 9–16; Table 3) that protects the structure-providing water molecule from the external solvent by strong shielding.

Overall, the compounds 2 to 20 suggest that the CK2β-competitive effect of a ligand is stronger the better it can recruit a water molecule and place it at the enzyme/ligand interface in such a way that it optimally forms cross-linking hydrogen bonds. If this is true, it should be possible to increase the affinity of this class of CK2β antagonists by optimising the Pro5 substituent: either by improving the environment of the critical water molecule so that it is even better harboured than in compound 12 or by replacing it functionally by a chemical extension of the ligand.

## Conclusion

The characteristic heterotetrameric quaternary structure of protein kinase CK2, which is unique among eukaryotic protein kinases, provides an opportunity to functionally impair the enzyme by interfering with the protein–protein interaction between the CK2α and CK2β subunits. Through high-throughput screening, cyclic pentapeptides with two non-proteinogenic amino acids [(*S*)-β-phenylalanine and a derivatized proline) were discovered to have the ability to disturb the assembly of CK2α and CK2β, as well as to disrupt a pre-formed CK2α/CK2β complex. In this functionality, these novel CK2β antagonists show a certain preference in favour of CK2α over its isoenzyme CK2α′. Crystal structure analyses provide evidence that this selectivity is due to the structural adaptability of the β4β5 loop in CK2α, a distinctive feature from CK2α′; they also suggest that the affinity of the peptides to the enzyme can be improved by optimizing the substituent of the derivatized proline residue.

## Author contributions

Conceptualisation: K. N. and M. P.; investigation: C. W., S. E., M. L., D. L., E. S., E. K., L. K., M. S., R. B., S. S., C. F., A. O., M. N. and C. G.; formal analysis: C. W., S. E., M. L., D. L., E. S., E. K., L. K., M. S., R. B., S. S., C. F., A. O., M. N., C. G., K. N. and M. P.; visualisation: S. E. and K. N.; data curation: M. N., C. G., K. N. and M. P.; resources: D. F., C. G., K. N. and M. P.; project administration: K. N. and M. P.; supervision: C. G., K. N. and M. P.; funding acquisition: K. N. and M. P.; writing – original draft: C. W., S. E., K. N., and M. P.; writing – review & editing: all authors.

## Conflicts of interest

There are no conflicts to declare.

## Supplementary Material

CB-007-D6CB00095A-s001

## Data Availability

Crystallographic data of the three complex structures of this article have been deposited at the PDB under the accession codes 9FBI (CK2α′^Cys336Ser^/cpd. 12), 9FBM (CK2α^1–335^/cpd. 12) and 9FBL (CK2α^1–335^/cpd. 15) and can be obtained from https://doi.org/10.2210/pdb9FBI/pdb, https://doi.org/10.2210/pdb9FBM/pdb and https://doi.org/10.2210/pdb9FBL/pdb. The raw X-ray diffraction data are available at the ESRF *via*https://doi.org/10.15151/esrf-dc-2428907319 (CK2α′^Cys336Ser^/cpd. 12), https://doi.org/10.15151/ESRF-DC-2427484222 (CK2α^1–335^/cpd. 12) and https://doi.org/10.15151/ESRF-DC-2428907308 (CK2α^1–335^/cpd. 15). Further data supporting the results and conclusions of this article, as well as the complete Experimental section, can be found in the supplementary information (SI). Supplementary information is available. See DOI: https://doi.org/10.1039/d6cb00095a.
