# Supplementary material for: Discovery and characterization of a novel class of cyclic peptidic compounds inhibiting the subunit interaction of the protein kinase CK2α2β2 holoenzyme
Source: RSC Chem Biol. 2026 Jun 23;7(9):1730–42. doi: 10.1039/d6cb00095a (PMC13353061; doi:10.1039/d6cb00095a)
Supplement: Supplementary file 1 [file CB-007-D6CB00095A-s001.pdf]

Supplementary Information (SI) for RSC Chemical Biology.
This journal is © The Royal Society of Chemistry 2026

Electronic Supporting Information

Discovery and characterization of a novel class of cyclic peptidic compounds inhibiting the subunit interaction of the protein kinase CK2$\alpha_2\beta_2$ holoenzyme

Christian Werner,[#a] Sophia Eimermacher,[#bc] Miriam Lauwers,[c] Dirk Lindenblatt,[a] Esra Seymen,[c] Ekaterina Kulko,[c] Leonard Klein,[b] Michaela Steinkrüger,[c] Robin Baumann,[b] Sarah Salamon,[c] Cora Fried,[c] Dietmar Fischer,[c] Andreas Oder,[d] Martin Neuenschwander,[d] Claudia Götz,[e] Karsten Niefind *[a] and Markus Pietsch *[bc]

a. Universität zu Köln, Department für Chemie und Biochemie, Institut für Biochemie, Zülpicher Straße 47, D-50674 Köln, Germany. E-mail: karsten.niefind@uni-koeln.de
b. Technische Hochschule Köln, Fakultät für Angewandte Naturwissenschaften, Campusplatz 1, D-51379 Leverkusen, Germany. E-mail: markus.pietsch@th-koeln.de
c. Universität zu Köln, Medizinische Fakultät und Uniklinik Köln, Zentrum für Pharmakologie, Institute I&II für Pharmakologie, Gleueler Straße 24, D-50931 Köln, Germany.
d. Leibniz-Forschungsinstitut für Molekulare Pharmakologie im Forschungsverbund Berlin e.V. (FMP), Screening Unit, Campus Berlin-Buch, Robert-Roessle-Straße 10, D-13125 Berlin, Germany.
e. Universität des Saarlandes, Medizinische Biochemie und Molekularbiologie, Kirrbergerstraße, Gebäude 44, D-66421 Homburg, Germany.

Common first authors.

Table of content

1. Figures and Tables mentioned in the article 3

2. Experimental Section 19

2.1 Plasmid construction 19

2.2 Protein expression and purification 19

2.3 Synthesis of Pc peptides 21

2.4 High-throughput screening of compound library... 22

2.5 Fluorescence anisotropy assay 23

2.6 Native PAGE for analysis of protein-protein interactions............................ 23

2.7 Analysis of protein-protein interactions by FRET.. 24

2.8 CK2-catalyzed substrate phosphorylation 27

2.9 Crystallization and crystal structure determination 28

3. References 30

1. Figures and Tables mentioned in the article

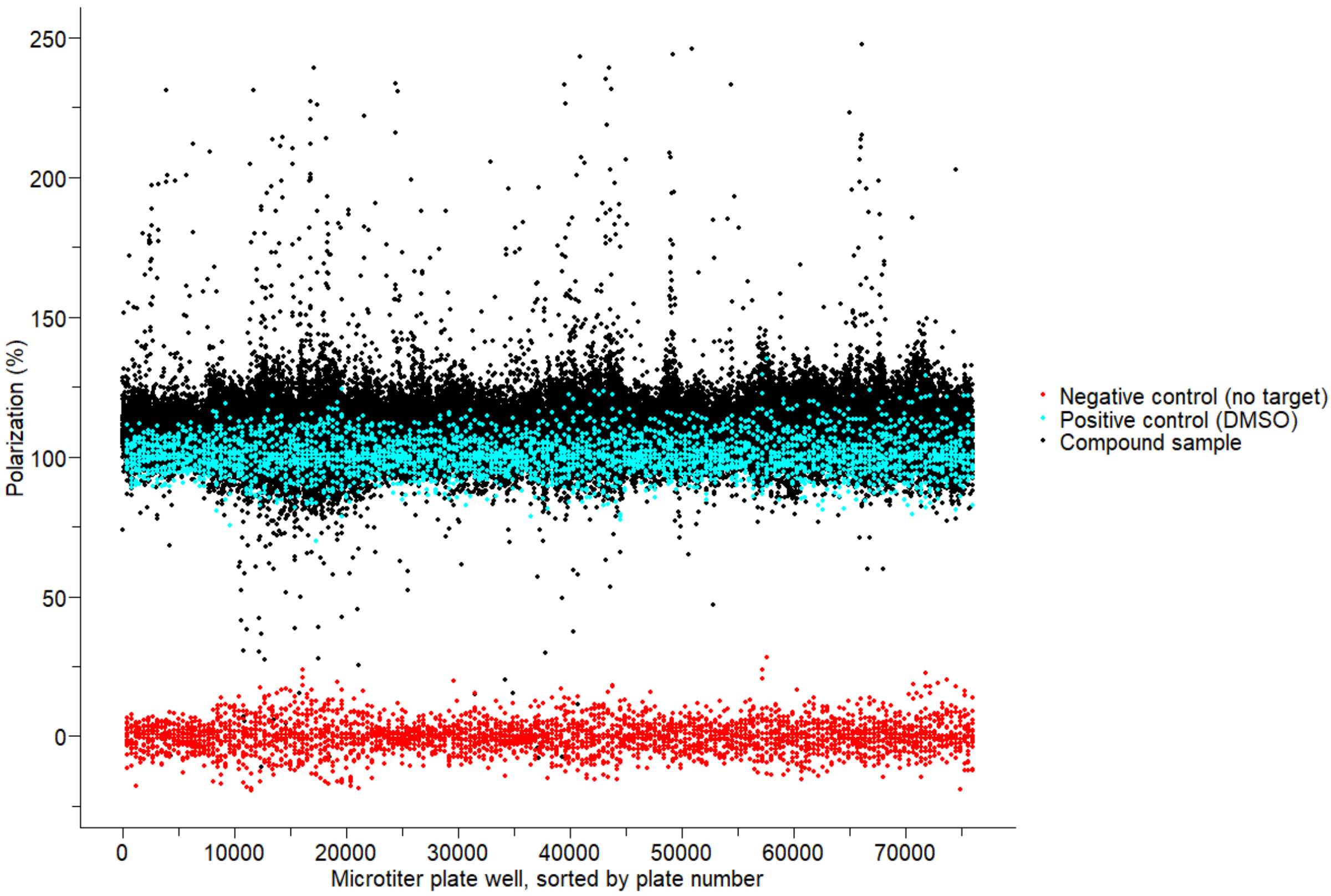

Figure S1. Scatter chart of percentage fluorescence polarization values of positive controls (CF-Ahx-Pc, CK2α$^{1\text{-}335}$, and DMSO), negative controls (CF-Ahx-Pc and DMSO) and compound samples (CF-Ahx-Pc, CK2α$^{1\text{-}335}$, and 20 µM compound) from the primary screen of 67,584 compounds on 198 microplates (384-well format).

Table S1. Evaluation of hits identified in the high-throughput screening (Figure 2) by determination of the value IC_{50} for the displacement of CF-Ahx-Pc from CK2α$^{1\text{-}335}$. The cyclic tridecapeptide I-Pc (Fig. 1b, K_i = 0.158 ± 0.051 μM, mean value ± SEM, n = 3)[1] served as a reference competitor to CF-Ahx-Pc. General structure of the nine cyclic pentapeptides **2**, **4**, **5**, **8**, **12**, **14**, **15**, **18** and **20** was as follows:

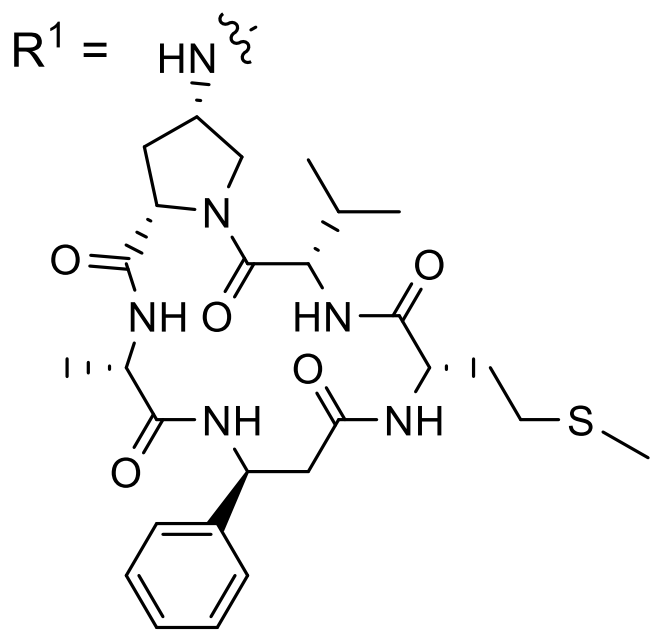

compd	structure	IC_{50} (μM)[a]	lower plateau[b] (*BOTTOM*, %)
**I-Pc**		0.885 (K_i = 0.0669 μM)[c]	2.79
FMP-109557 (**5**)	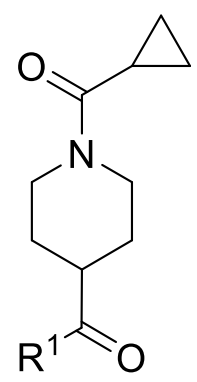	15.8	13.6
FMP-109594 (**4**)	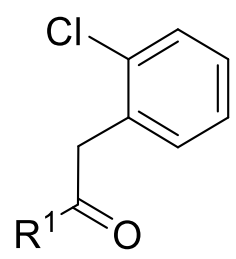	19.1	19.3
FMP-109735 (**20**)	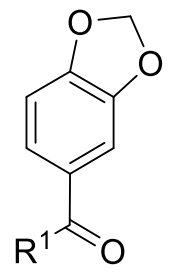	15.9	1.79×10^{-9}
FMP-109788 (**2**)	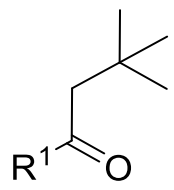	13.6	34.1

FMP-109957 (**12**)	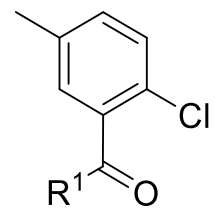	2.67	4.28×10^{-7}
FMP-109959 (**8**)	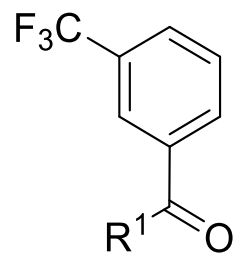	9.13	7.69×10^{-6}
FMP-109969 (**18**)	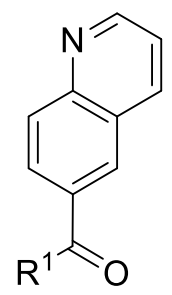	9.88	17.4
FMP-110025 (**15**)	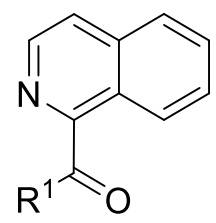	15.2	4.80×10^{-7}
FMP-110060 (**14**)	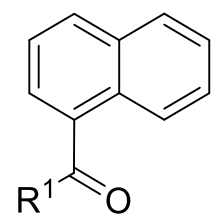	3.73	2.58×10^{-7}
FMP-203351	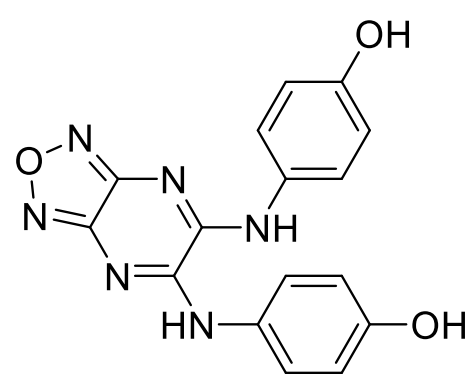	3.04	52.3[d]

[a] IC_{50} values were obtained in a single duplicate experiment in 25 mM Tris-HCl, pH 8.5, 500 mM NaCl, 2% (v/v) DMSO, 0.05% (v/v) Tween 20 using 1 µM of $CK2\alpha^{1-335}$, 0.1 µM of CF-Ahx-Pc, and 0.0391-20.0 µM (I-Pc) or 0.0488-50.0 µM (FMP compounds) of competitor. IC_{50} values were calculated according to the four-parameter equation setting the value of *BOTTOM* to larger than zero.

[b] The value of the upper plateau (*TOP*) obtained by the four-parameter equation was 94.8-105%.

[c] The value of K_i was calculated according to Nikolovska-Coleska et al.[2] using a K_D value of 0.602 µM, obtained in a single duplicate experiment with 0.0391-20.0 µM of $CK2\alpha^{1-335}$ and 0.1 µM of CF-Ahx-Pc in the absence of DMSO, and the "K_i calculator" (http://www.umich.edu/~shaomengwanglab/software/ calc_ki/index.html). When using 3 µM instead of 1 µM of $CK2\alpha^{1-335}$ in the displacement assay, an IC_{50} value of 3.67 µM was obtained that gave a value of K_i = 0.191 µM. One sample t-tests comparing the reference K_i value of 0.158 ± 0.051 µM (n = 3) for I-Pc with either of the two K_i values of 0.0669 and 0.191 µM yielded no significant differences with P values of 0.2160 and 0.5839, respectively.

[d] FMP-203351 showed insufficient displacement of CF-Ahx-Pc from $CK2\alpha^{1-335}$ (less than 50% even at high compound concentrations) and, therefore, was not further investigated herein.

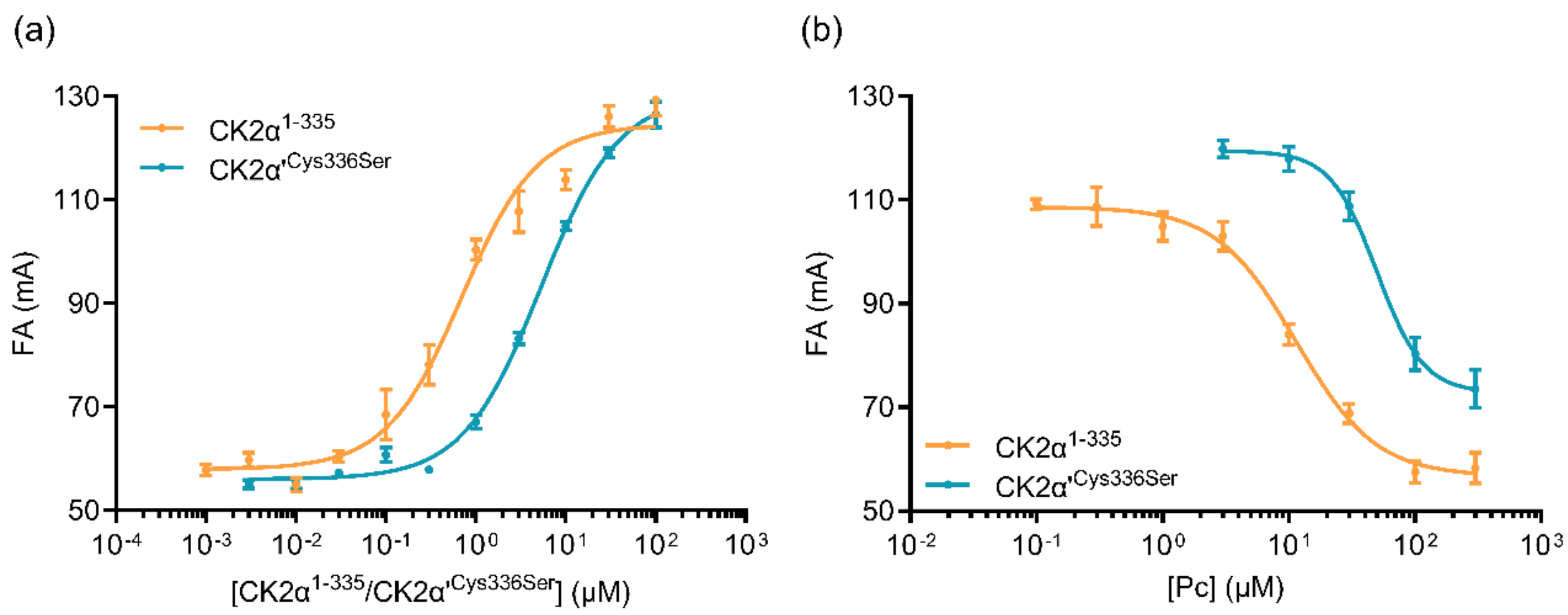

Figure S2. Binding of CF-Ahx-Pc to CK2α$^{1-335}$ or CK2α'^{Cys336Ser} (a) and displacement of CF-Ahx-Pc by unlabelled Pc (b) investigated on a Synergy™ 2 plate reader (BioTek, USA). All measurements were done in 25 mM Tris, 500 mM NaCl, pH 8.5 using 0.1 µM of CF-Ahx-Pc and 0.001-100 µM CK2α$^{1-335}$ or 0.003-100 µM CK2α'^{Cys336Ser} for (a). (b) Experiments were done with 0.1 µM CF-Ahx-Pc and either 3 µM CK2α$^{1-335}$ and 0.1-300 µM Pc or 30 µM CK2α^{Cys336Ser} and 3-300 µM Pc. CK2α$^{1-335}$ was investigated in a total volume of 50 µL in a 384-well plate format, whereas a total volume of 200 µL in a 96-well plate format was used for CK2α'^{Cys336Ser}. All data points shown are mean values ± SEM of three to four separate experiments, each performed in duplicate or triplicate. Analysis of data in (a) by nonlinear regression according to Hochscherf et al.[3] resulted in K_D values of 0.659 ± 0.137 µM for CK2α$^{1-335}$ and 5.27 ± 0.60 µM for CK2α'^{Cys336Ser}, respectively. Displacement of CF-Ahx-Pc by Pc in (b) was quantified by means of the four-parameter equation, resulting in IC_{50} values of 11.6 ± 1.7 µM and 50.1 ± 3.1 µM for CK2α$^{1-335}$ and CK2α'^{Cys336Ser}, respectively. Coefficients of determination for all curve fits are provided in Table S4. K_i values of 1.7 ± 0.3 µM for CK2α$^{1-335}$ and 3.6 ± 0.5 µM for CK2α'^{Cys336Ser} were calculated according to Nikolovska-Coleska et al.[2] using the K_D values determined in (a) and the "K_i calculator" (http://www.umich.edu/~shaomengwanglab/software/calc_ki/index.html).

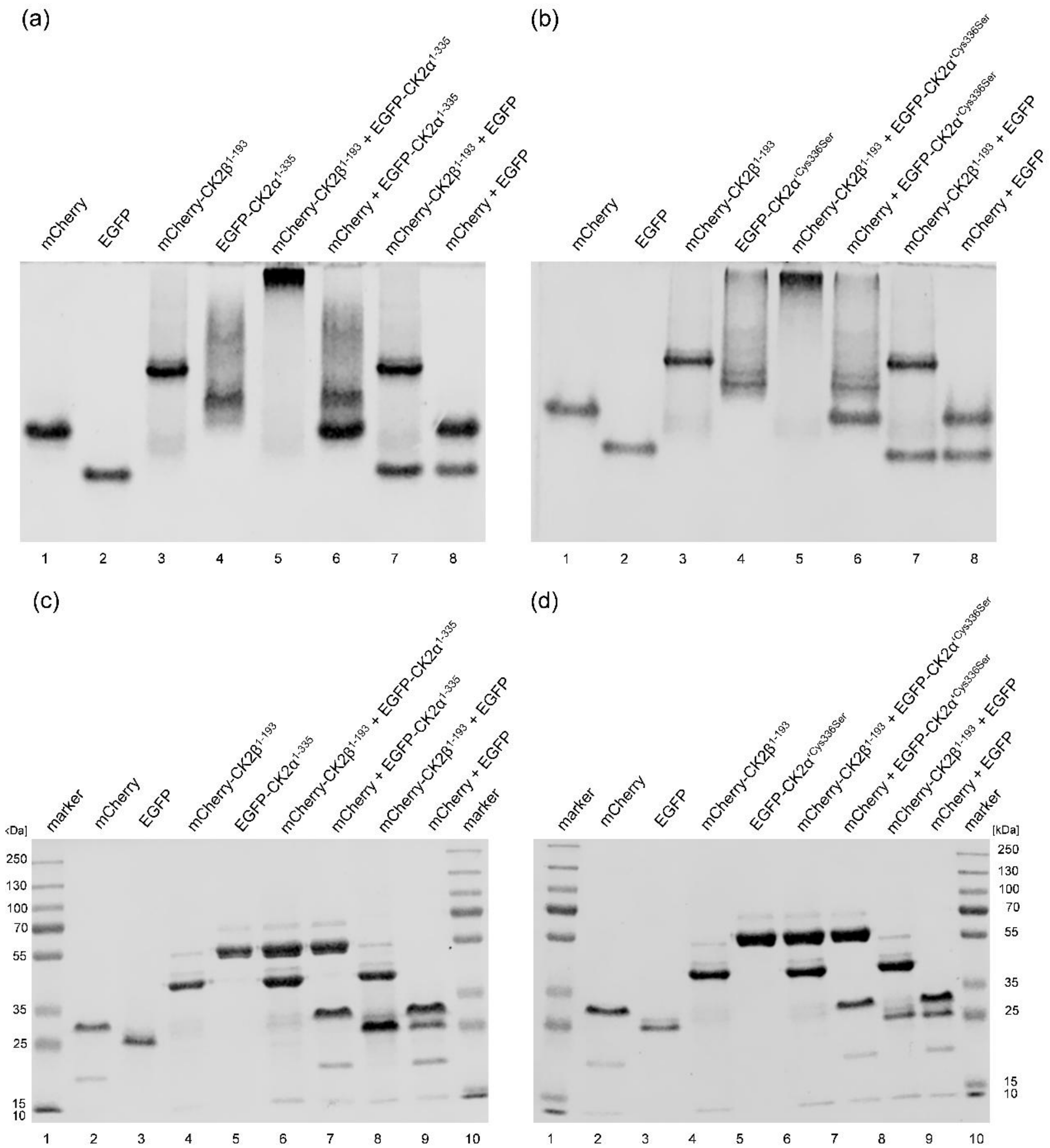

Figure S3. Protein-protein interactions visualized on Coomassie Brilliant Blue G250-stained 10% native PAGE gels (a, b) and on 10% SDS-PAGE gels (c, d). For all gels, 20 µM of the respective protein (lane 1-4 in (a, b) and lane 2-5 in (c, d)) or protein combinations (lane 5-8 in (a, b) and lane 6-9 in (c, d)) were incubated at room temperature for 15 min in 25 mM Tris, 500 mM NaCl, pH 8.5 containing either native loading buffer (a, b) or SDS loading buffer (c, d). A volume of 5 µL of the protein mixtures and 3 µL of the marker PageRuler^{TM} Plus Prestained Protein Ladder (lanes 1 and 10 in (c, d)) were loaded onto the gels and electrophoresis was performed at a constant voltage of 80 V for the native gels (a, b) or 120 V for the SDS gels (c, d). After staining, the gels were scanned on a LI-COR Odyssey Fc Imager (700 nm-channel).

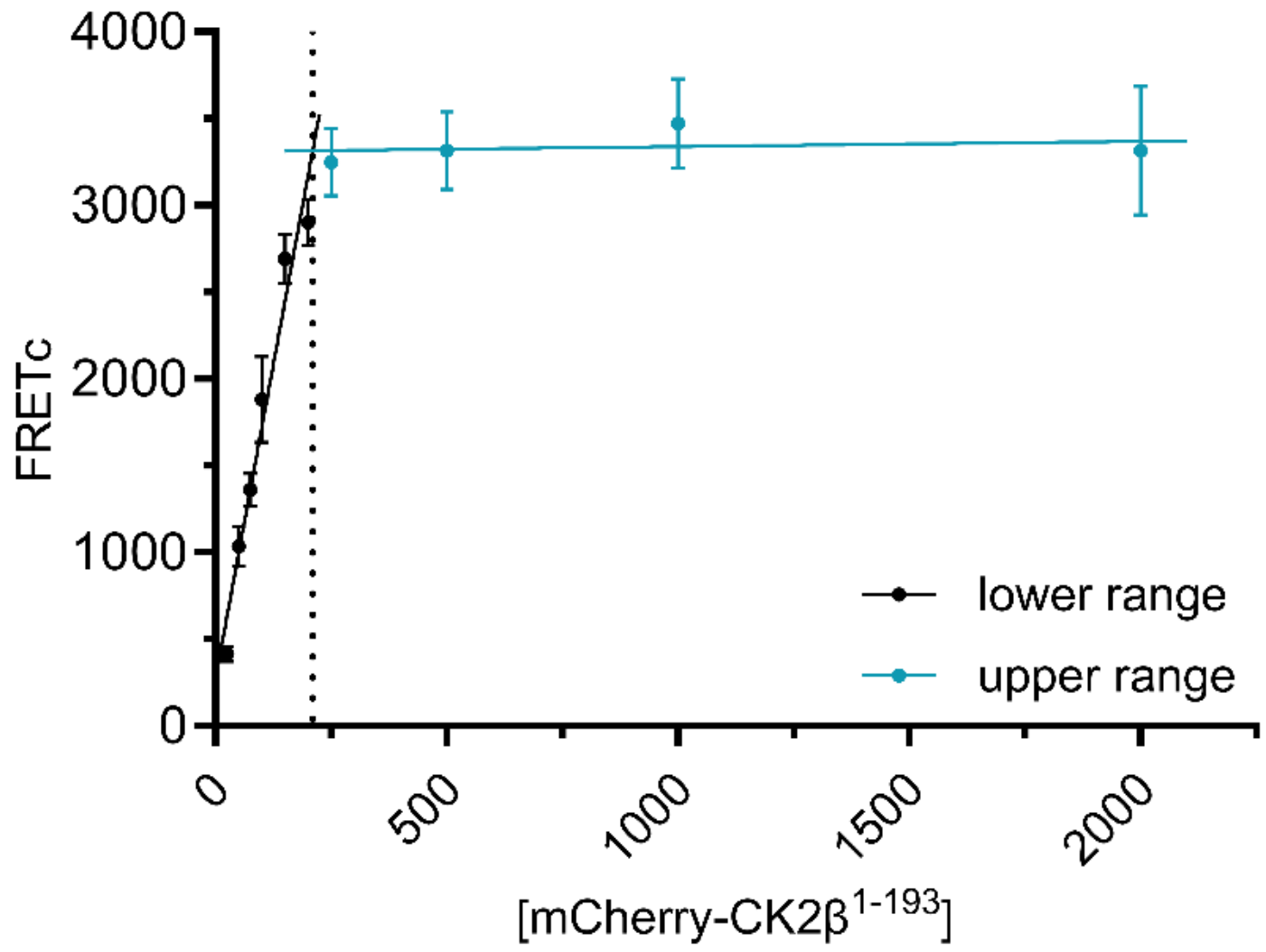

Figure S4. Titration of EGFP-CK2α$^{1\text{-}335}$ with mCherry-CK2β$^{1\text{-}193}$. A fixed concentration of EGFP-CK2α$^{1\text{-}335}$ (200 nM) was incubated with mCherry-CK2β$^{1\text{-}193}$ (25-2000 nM) for 30 min at room temperature before FRETc was determined using an Infinite® M1000 PRO plate reader (Tecan Group, Switzerland). Three separate experiments were performed in duplicate in a total volume of 50 µL on a 384-well plate. Conditions were as follows: 25 mM Tris, 500 mM NaCl, pH 8.5, 2% (v/v) DMSO, 0.05% (v/v) Tween 20. Data points below (black) and above (cyan) the mCherry-CK2β$^{1\text{-}193}$ concentration of 200 nM were subjected to linear regression. The mCherry-CK2β$^{1\text{-}193}$ concentration able to bind to 200 nM of EGFP-CK2α$^{1\text{-}335}$ (x-value of the intersection point represented by a dashed line) was calculated to 210 ± 4 nM by the equation $x = (n_2 - n_1)/(m_1 - m_2)$, with m and n representing the slopes and the intercepts of the lower and upper range, respectively. Coefficients of determination for all curve fits are provided in Table S4.

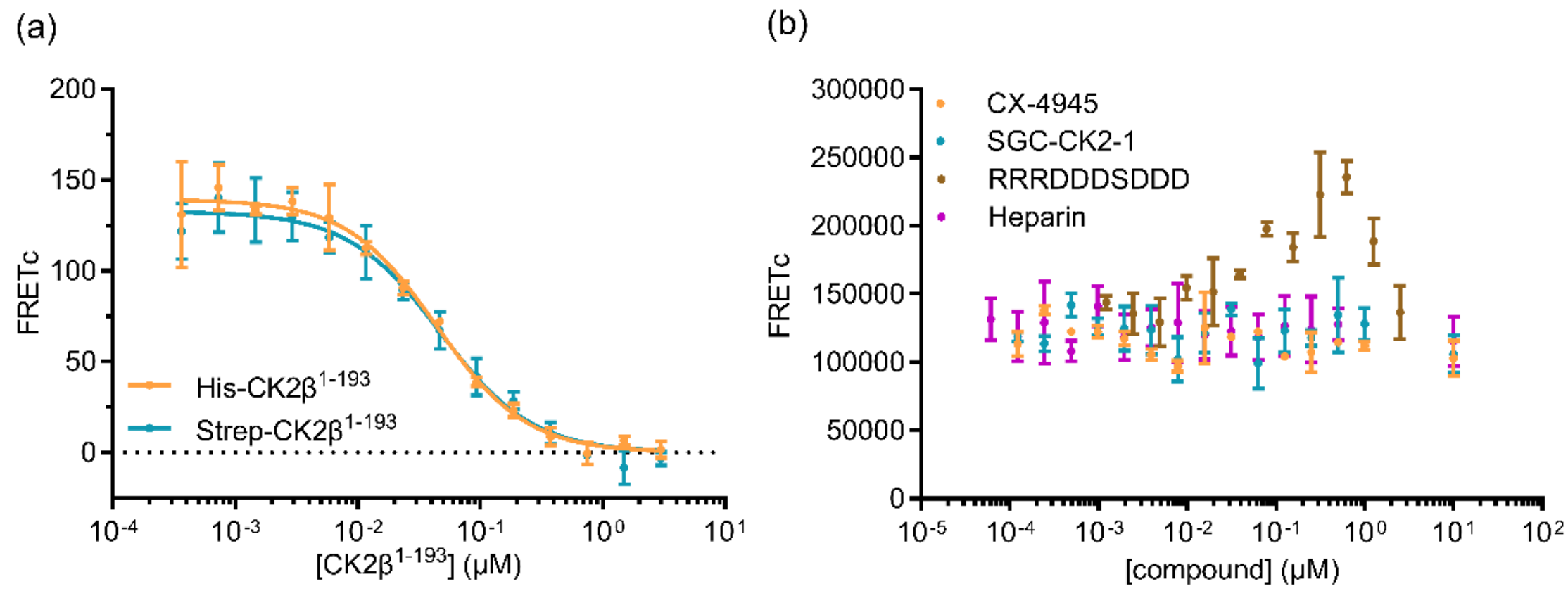

Figure S5. Investigations on the inhibition of binding of mCherry-CK2β$^{1\text{-}193}$ to EGFP-CK2α$^{1\text{-}335}$ by tagged CK2β$^{1\text{-}193}$ (a) and ATP or substrate site-directed compounds (b), respectively. Conditions were as follows: 25 mM Tris, 500 mM NaCl, pH 8.5, 2% (v/v) DMSO, 0.05% (v/v) Tween 20. Three separate experiments in duplicate were conducted in a total volume of 50 µL in the 384-well plate format using 30 nM of EGFP-CK2α$^{1\text{-}335}$ and 50 nM of mCherry-CK2β$^{1\text{-}193}$. Displacement of mCherry-CK2β$^{1\text{-}193}$ was investigated in the presence of 3.66×10^{-4}-3 µM His-CK2β$^{1\text{-}193}$ or Strep-CK2β$^{1\text{-}193}$ using an Infinite® M1000 PRO plate reader (Tecan Group, Switzerland) (a) or with 1.2×10^{-4}-10 µM of CX-4945 or SGC-CK2-1, 0.0012-10 µM of RRRDDDSDDD and 6×10^{-5}-10 µM Heparin, respectively, on a Synergy™ 2 plate reader (BioTek, USA) (b). IC_{50} values in (a) were calculated according to the four-parameter equation, yielding 0.0444 ± 0.0088 µM and 0.0578 ± 0.0095 µM for His-CK2β$^{1\text{-}193}$ and Strep-CK2β$^{1\text{-}193}$, respectively. Coefficients of determination for all curve fits are provided in Table S4. K_i values of 0.0022 ± 0.0007 µM for His-CK2β$^{1\text{-}193}$ and 0.0040 ± 0.0009 µM for Strep-CK2β$^{1\text{-}193}$ were obtained by applying K_D values of 3.41 nM (SEM: 0.41 nM) and 4.43 nM (SEM: 1.17 nM), respectively, obtained in two independent sets of three triplicate experiments for the binding of mCherry-CK2β$^{1\text{-}193}$ to EGFP-CK2α$^{1\text{-}335}$, in the "K_i calculator" (http://www.umich.edu/~shaomengwanglab/software/calc_ki/index.html).

Table S2. Characterization of cyclic pentapeptides with regard to binding to CK2α$^{1\text{-}335}$ and disturbing the interaction of mCherry-CK2β$^{1\text{-}193}$ with EGFP-CK2α$^{1\text{-}335}$ and EGFP-CK2α'^{Cys336Ser}, respectively.

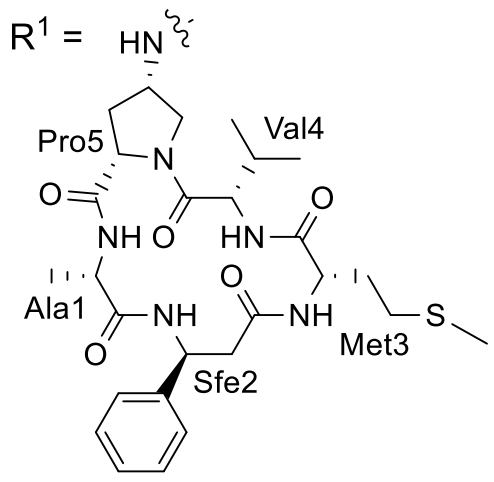

1-20

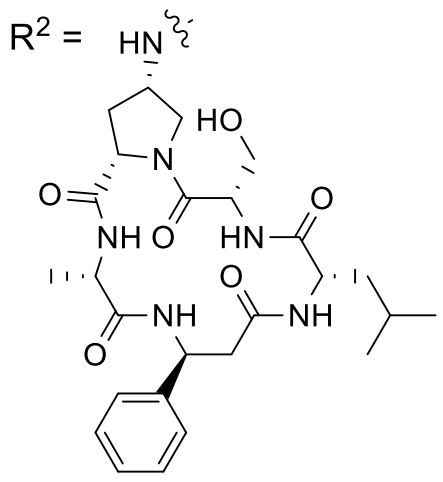

21-25

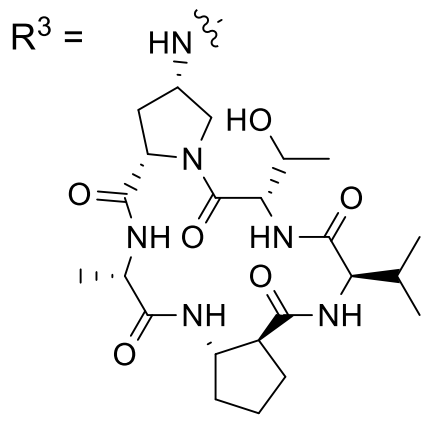

26

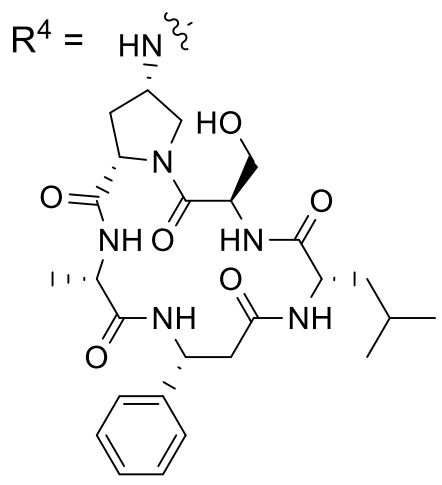

27, 28

		CK2α$^{1\text{-}335}$		EGFP-CK2α$^{1\text{-}335}$		EGFP-CK2α'^{Cys336Ser}	
**compd**	**structure**	**IC_{50} (µM)**	**K_i (µM)^a**	**IC_{50} (µM)**	**K_i (µM)^a**	**IC_{50} (µM)**	**K_i (µM)^a**
**1**	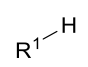	> 300^b	> 70	~ 200^c	~ 15	> 200^b	> 30
**2**	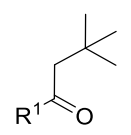	35.7 ± 3.3	8.0 ± 0.8	33.6 ± 1.4	2.5 ± 0.1	> 100^b	> 15
**3**	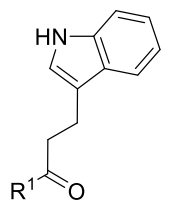	> 100^b	> 23	~ 100^c	~ 7.5	> 100^b	> 15
**4**	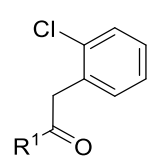	89.4 ± 12.8	21 ± 3	42.6 ± 12.4	3.2 ± 0.9	> 20^b	> 3.0
**5**	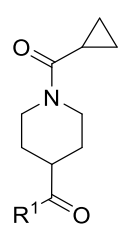	50.0 ± 7.4	11 ± 2	16.0 ± 4.2	1.6 ± 0.4	96.8 ± 10.9	14 ± 2
**6**	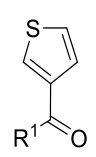	43.3 ± 0.3	9.8 ± 0.1	32.7 ± 10.2	2.5 ± 0.8	110 ± 20	16 ± 3
**7**	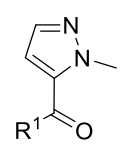	62.1 ± 8.8	14 ± 2	103 ± 9	7.7 ± 0.7	> 20^b	> 3.0
**8**	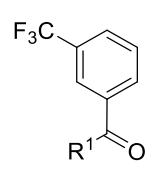	22.4 ± 2.6	4.9 ± 0.6	15.8 ± 1.9	1.2 ± 0.1	58.6 ± 9.1	8.7 ± 1.4
**9**	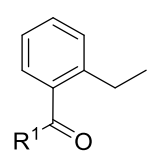	10.9 ± 1.6	2.2 ± 0.4	10.6 ± 2.8	0.80 ± 0.21	41.3 ± 9.5	6.1 ± 1.4
**10**	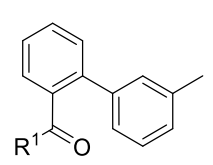	12.4 ± 0.6	2.5 ± 0.1	7.83 ± 2.42	0.59 ± 0.18	> 20^b	> 3.0
**11**	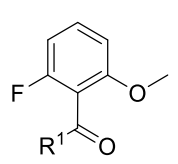	11.0 ± 0.6	2.2 ± 0.2	6.56 ± 0.46	0.49 ± 0.03	34.5 ± 7.1	5.1 ± 1.1

**12**	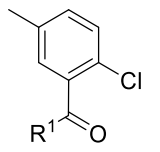	10.2 ± 2.3	2.0 ± 0.5	7.60 ± 2.06	0.57 ± 0.16	30.0 ± 6.1	4.5 ± 0.9
**13**	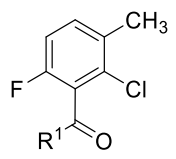	5.50 ± 0.52	0.92 ± 0.12	1.99 ± 0.18	0.15 ± 0.01	16.3 ± 2.0	2.4 ± 0.3
**14**	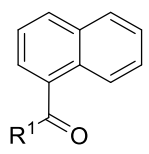	5.79 ± 0.99	0.99 ± 0.23	4.33 ± 0.97	0.32 ± 0.07	57.3 ± 5.7	8.5 ± 0.8
**15**	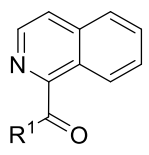	13.3 ± 1.8	2.7 ± 0.4	9.88 ± 1.17	0.74 ± 0.09	> 200[b]	> 30
**16**	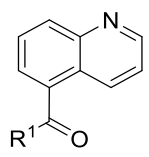	11.2 ± 1.6	2.4 ± 0.4	13.4 ± 3.4	1.0 ± 0.3	74.6 ± 19.7	11 ± 3
**17**	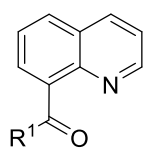	39.0 ± 9.2	9.3 ± 2.3	20.3 ± 8.2	1.5 ± 0.6	> 200[b]	> 30
**18**	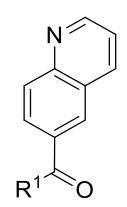	~ 100[c]	~ 23	21.0 ± 3.1	2.1 ± 0.3	> 100[b]	> 15
**19**	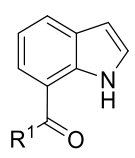	22.0 ± 0.3	5.1 ± 0.1	22.5 ± 2.0	1.7 ± 0.2	> 100[b]	> 15
**20**	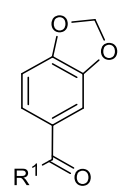	32.9 ± 11.4	7.3 ± 2.7	30.5 ± 2.7	2.3 ± 0.2	117 ± 28	17 ± 4
**21**	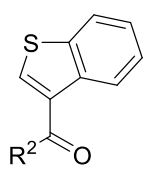	> 100[b]	> 23	> 100[b]	> 7.5	> 100[b]	> 15
**22**	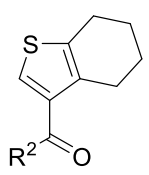	> 100[b]	> 23	> 100[b]	> 7.5	> 100[b]	> 15
**23**	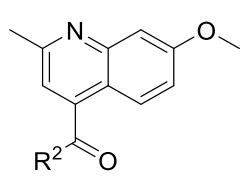	> 100[b]	> 23	> 100[b]	> 7.5	> 100[b]	> 15
**24**	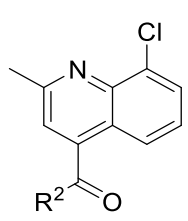	> 300[b]	> 70	> 200[b]	> 15	> 100[b]	> 15
**25**	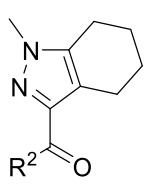	> 300[b]	> 70	> 200[b]	> 15	> 100[b]	> 15
**26**	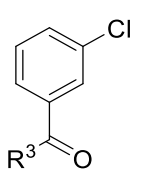	> 300[b]	> 70	> 200[b]	> 15	> 100[b]	> 15
**27**	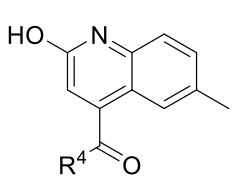	> 300[b]	> 70	> 200[b]	> 15	> 100[b]	> 15
**28**	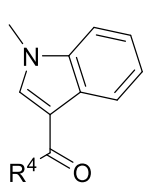	> 300[b]	> 70	> 200[b]	> 15	> 100[b]	>15

[a] Dissociation constants K_i are the same as those in Table 3 and were calculated form the respective IC_{50} values (mean values ± SEM, n = 3-4) obtained on a Synergy™ 2 plate reader (BioTek, USA) by means of the "K_i calculator" (http://www.umich.edu/~shaomengwanglab/software/calc_ki/index.html) using K_D values of protein preparations of 0.609 µM (CK2α$^{1-335}$), 3.28 nM (EGFP-CK2α$^{1-335}$) and 7.90 nM (EGFP-CK2α'^{Cys336Ser}), respectively. [b] Less than 50% inhibition was observed at the highest compound concentration investigated. [c] About 50% inhibition was observed at the highest compound concentration investigated.

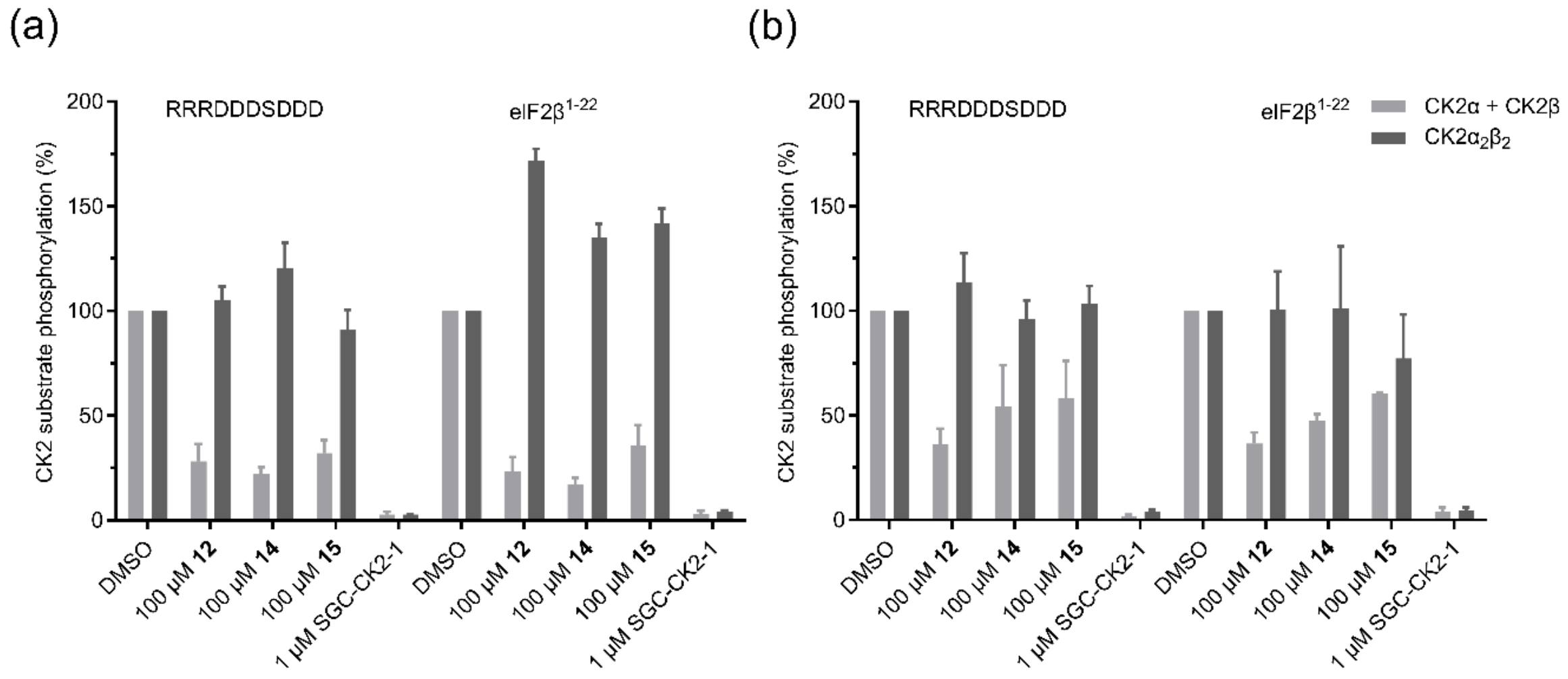

Figure S6. Determination of the kinase activity of full-length CK2α in presence of full-length CK2β (light grey bars) and $CK2\alpha_2\beta_2$ holoenzyme (dark grey bars) by analysing the phosphorylation of the peptides RRRDDDSDDD (class I substrate, left) and $eIF2\beta^{1\text{-}22}$ (class III substrate, right) by γ-^{32}P-ATP starting either directly (a) or after 10 min of incubation of inhibitors with protein at room temperature (b). Measurements were done using the following conditions: 50 mM Tris-HCl, pH 7.5, 150 mM NaCl, 5 mM $MgCl_2$, 1 mM DTT. For experiments using the individual subunits (light grey bars), a volume of 1 µL of 5 mM cyclic pentapeptide or 0.05 mM SGC-CK2-1 in DMSO was added to CK2α (final concentration of 90 nM). The same concentration of CK2β was added to the mixture after 1-2 min of incubation at room temperature. For experiments with the CK2 holoenzyme, a final concentration of 10 nM $CK2\alpha_2\beta_2$ was used. The reaction was started either immediately or after 10 min of incubation at room temperature by addition of a mixture of substrate peptide (0.19 mM RRRDDDSDDD or $eIF2\beta^{1\text{-}22}$) and ATP (20 µCi mL^{-1} γ-^{32}P-ATP and 50 µM ATP). Measurements in the presence of DMSO and the reported ATP site-directed CK2 inhibitor SGC-CK2-1[4] served as negative and positive controls, respectively. Depicted are mean values ± SEM of five (a) or two (b) experiments, each performed in duplicate.

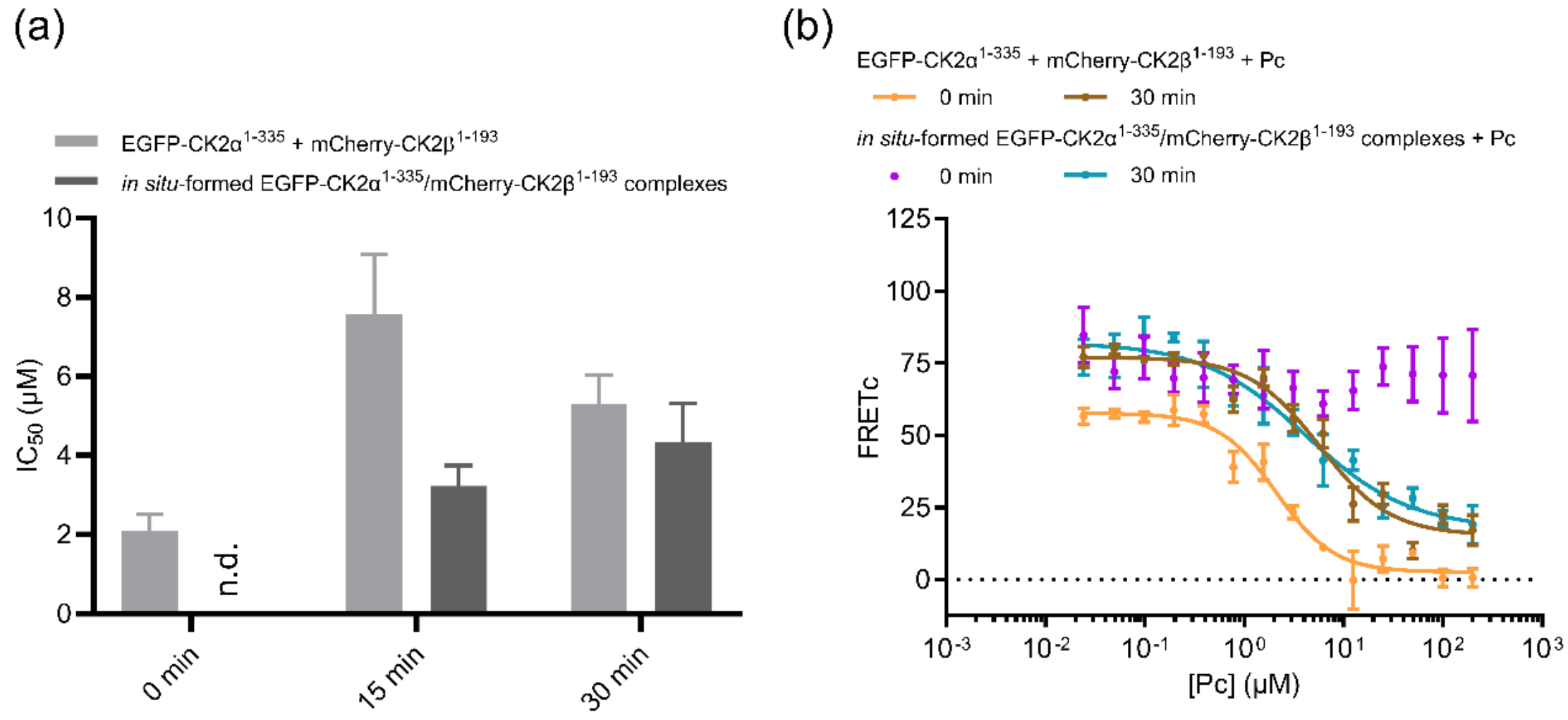

Figure S7. Inhibition of binding of mCherry-CK2β$^{1-193}$ to EGFP-CK2α$^{1-335}$ and dissociation of pre-formed EGFP-CK2α$^{1-335}$/mCherry-CK2β$^{1-193}$ complexes by Pc. Results shown in Figure S7a have also been provided in Figure 5e. Three separate experiments were performed in duplicate using the following conditions: 25 mM Tris, 500 mM NaCl, pH 8.5, 2% (v/v) DMSO, 0.05% (v/v) Tween 20. Experiments were done in a total volume of 50 µL using a 384-well-plate. Either the individual subunits (75 nM each) or a mixture of both subunits (37.5 nM each) were incubated for 30 min at 30 °C. After incubation, the subunits or the mixture were added to Pc to obtain a final concentration of the individual subunits or the complex of 30 nM each and displacement of mCherry-CK2β$^{1-193}$ from EGFP-CK2α$^{1-335}$ was investigated in the presence of 0.024-200 µM Pc using an Infinite® M1000 PRO plate reader (Tecan Group, Switzerland). FRET was investigated either directly after addition of the subunits and the mixture, respectively, or after an incubation period of 15 or 30 min. IC_{50} values in (a) were calculated according to the four-parameter equation, yielding 2.08 ± 0.43 µM (0 min), 7.56 ± 1.53 µM (15 min) and 5.30 ± 0.73 µM (30 min) for the individual subunits (light grey bars). Disruption of *in situ*-formed EGFP-CK2α$^{1-335}$/mCherry-CK2β$^{1-193}$ complexes by Pc (dark grey bars in (a)) was quantified by the following IC_{50} values: 3.23 ± 0.52 µM (15 min), 4.33 ± 0.99 µM (30 min). Dose-response curves of the displacement of mCherry-CK2β$^{1-193}$ by Pc after 0 and 30 min are shown in (b). No disruption of the EGFP-CK2α$^{1-335}$/mCherry-CK2β$^{1-193}$ complex was observed when the pre-incubated mixture was added to Pc and FRET was immediately investigated

after addition (purple curve, (b)). Coefficients of determination for all curve fits are provided in Table S4.

Table S3. X-ray diffraction data and refinement statistics of CK2α$^{1-335}$ and CK2α'^{Cys336Ser} complex crystals

Crystallized complex	CK2α'^{Cys336Ser} with cpd. **12**	CK2α$^{1-335}$ with cpd. **12**	CK2α$^{1-335}$ with cpd. **15**
PDB code	9FBI	9FBM	9FBL
DOI access of raw X-ray diffraction data	10.15151/ESRF-ES-1007017045	10.15151/ESRF-ES-1317816410	10.15151/ESRF-ES-1118696478
X-ray diffraction data quality			
Wavelength	0.8856 Å	0.9677 Å	0.8856 Å
Synchrotron (beamline)	ESRF (ID30B)[5]	ESRF (ID30A-3)[6]	ESRF (ID23-1)[7]
Space group	$P2_1$	$P2_12_12$	$P2_12_12$
Cell dimensions			
a, b, c [Å]	46.452, 71.068, 102.878	78.016, 208.360, 76.718	77.952, 206.56, 76.772
α, β, γ [°]	90, 91.967, 90	90, 90, 90	90, 90, 90
Resolution [Å] (highest resolution shell)#	58.462 - 1.161 (1.329-1.161)	76.718 – 2.054 (2.182–2.054)	72.931 – 2.176 (2.403–2.176)
R_{sym} [%]#	8.1(127.5)	56.2 (664.1))	47.9 (839.7)
CC1/2#	0.906 (0.535)	0.994 (0.563)	0.990 (0.502)
Signal-to-noise ratio (I/σ_I)#	7.2 (1.8)	9.6 (1.5)	8.1 (1.6)
No. of unique reflections#	119426 (5971)	68956 (3448)	39662 (1983)
Completeness (spherical) [%]#	52.0 (7.8)	87.5 (26.8)	60.2 (11.9)
Completeness (ellips.) [%]§,#	86.0 (62.0)	94.7 (48.3)	93.6 (68.1)
Multiplicity#	4.9 (6.3)	35.3 (37.4)	17.6 (17.7)
Wilson-plot B-factor [Å^2]	13.04	21.56	38.94
Refinement and structure quality			
No. of refl. for R_{work}/R_{free}	118163/1205	67912/1023	38434/1186
R_{work}/R_{free} [%]	0.1452/0.1847	0.1847/0.2388	0.2068/0.2644
Protomers per asymmetric unit	2	3	3
No. of non-H-atoms	6236	9450	8840
Protein	5634	8435	8397
Ligand/ion	117	226	246
Water	485	789	197
Average B-Factors [Å^2]	20.49	32.82	51.90
Protein	19.44	32.67	51.81
Ligand/ion	29.13	39.03	66.25
Water	30.67	32.65	37.87
RMS deviations			
Bond lengths [Å]	0.013	0.007	0.002
Bond angles [°]	1.16	0.90	0.48
Ramachandran plot			
Favored [%]	96.93	96.76	96.14
Allowed [%]	2.91	3.14	3.66
Outliers [%]	0.15	0.10	0.20

#Values in brackets refer to the highest resolution shell.
§According to anisotropic analysis with STARANISO[8]

Table S4. Coefficients of determination (R^2) for data analyses depicted in the main text and the Electronic Supporting Information. Mean values of R^2 ± SEM from three to four separate experiments, each performed in duplicate to triplicate, are listed.

Figure	Condition or compound	R^2
3a	w/ DMSO & Tween 20 (EGFP-CK2α$^{1-335}$)	0.935 ± 0.012
	w/ DMSO (EGFP-CK2α$^{1-335}$)	0.884 ± 0.051
	w/o DMSO & Tween (EGFP-CK2α$^{1-335}$)	0.903 ± 0.011
	w/ DMSO & Tween 20 (EGFP-CK2α'^{C336S})	0.931 ± 0.013
	w/ DMSO (EGFP-CK2α'^{C336S})	0.955 ± 0.010
	w/ Tween (EGFP-CK2α'^{C336S})	0.979 ± 0.004
	w/o DMSO & Tween (EGFP-CK2α'^{C336S})	0.949 ± 0.013
4	CK2β$^{1-193}$ (EGFP-CK2α$^{1-335}$)	0.884 ± 0.009
	I-Pc (EGFP-CK2α$^{1-335}$)	0.877 ± 0.023
	Pc (EGFP-CK2α$^{1-335}$)	0.961 ± 0.017
	Pc (EGFP-CK2α'^{C336S})	0.858 ± 0.011
5a	**12**	0.896 ± 0.011
	**13**	0.853 ± 0.029
	**14**	0.819 ± 0.011
	**15**	0.798 ± 0.009
5b	**12**	0.815 ± 0.038
	**13**	0.799 ± 0.017
	**14**	0.664 ± 0.074
	**15**	/
5c	**12**	0.966 ± 0.006
	**13**	0.976 ± 0.0002
	**14**	0.963 ± 0.003
	**15**	0.926 ± 0.019
5f	EGFP-CK2α$^{1-335}$ + mCherry-CK2β$^{1-193}$ + **12** (0 min)	0.862 ± 0.024
	EGFP-CK2α$^{1-335}$ + mCherry-CK2β$^{1-193}$ + **12** (30 min)	0.848 ± 0.031
	in situ-formed EGFP-CK2α$^{1-335}$/mCherry-CK2β$^{1-193}$ complexes + **12** (0 min)	/
	in situ-formed EGFP-CK2α$^{1-335}$/mCherry-CK2β$^{1-193}$ complexes + **12** (30 min)	0.884 ± 0.031
S2a	CK2α$^{1-335}$	0.959 ± 0.007
	CK2α'^{C336S}	0.988 ± 0.006
S2b	CK2α$^{1-335}$	0.954 ± 0.004
	CK2α'^{C336S}	0.983 ± 0.007
S4	Lower range	0.938 ± 0.012
	Upper range	0.236 ± 0.082
S5a	His-CK2β$^{1-193}$	0.929 ± 0.027
	Strep-CK2β$^{1-193}$	0.955 ± 0.008
S7b	EGFP-CK2α$^{1-335}$ + mCherry-CK2β$^{1-193}$ + Pc (0 min)	0.843 ± 0.020
	EGFP-CK2α$^{1-335}$ + mCherry-CK2β$^{1-193}$ + Pc (30 min)	0.867 ± 0.011
	in situ-formed EGFP-CK2α$^{1-335}$/mCherry-CK2β$^{1-193}$ complexes + Pc (0 min)	/
	in situ-formed EGFP-CK2α$^{1-335}$/mCherry-CK2β$^{1-193}$ complexes + Pc (30 min)	0.857 ± 0.010

2. Experimental Section

2.1 Plasmid construction

Genetic information of EGFP and mCherry embedded in the pIRES plasmid and lentiCRISPR v2 plasmid, respectively, was cloned into the vector pPSG IBA 105 (IBA-Lifesciences, Göttingen, Germany) using the Esp3I restriction sites. To generate the respective CK2 construct, genetic information of CK2α$^{1\text{-}335}$ (pET22b(+)), CK2α'^{Cys336Ser} (pET-28a(+), Gene Universal, Newark, DE, USA) or CK2β$^{1\text{-}193}$ (pT7-7) was cloned into the respective plasmid using BamHI and SacI for orientation. Successful cloning was verified by sequencing. The three CK2 constructs contained an N-terminal Twin-Strep-Tag followed by EGFP or mCherry, which is connected to the respective CK2 subunit via a GGDGGGGG and a GGGGGGGGEL linker, respectively. The fusion proteins are referred to as EGFP-CK2α$^{1\text{-}335}$, EGFP-CK2α'^{Cys336Ser} and mCherry-CK2β$^{1\text{-}193}$, respectively.

2.2 Protein expression and purification

For crystallization experiments, CK2α$^{1\text{-}335}$ and CK2α'^{Cys336Ser} – both provided with an N-terminal (His)$_6$-tag – were prepared as described by Werner et al.[9, 10]. N-terminal (His)$_6$-tagged CK2α$^{1\text{-}335}$, which was used for compound library screening and all FA experiments with that CK2 subunit, was expressed and purified as reported by Lindenblatt et al.[1].

(His)$_6$-taged CK2α'^{Cys336Ser} was prepared following Werner et al.[10] but with some alterations as detailed below. Cells expressing CK2α'^{Cys336Ser} were pre-cultured at 37 °C overnight in 50 mL of LB medium (10 g/L tryptone, 5 g/L yeast extract, 10 g/L NaCl) containing 100 µg/mL ampicillin. Main culture (600 mL) was treated as described but cells were grown at 37 °C for 3h instead at 18 °C for 18 h. Cells were then centrifuged (4 °C, 7000 × g) and washed with ice-cold 0.9% NaCl containing 0.2 mM PMSF and the pellet was stored at -80 °C. Cells were lysed as described but in the presence of 300 mM instead of 500 mM NaCl. Purification of CK2α'^{Cys336Ser} was done using 50 mM HEPES, pH 7.5 with 300 mM NaCl, 10 mM imidazole, 5% (v/v) glycerol 0.5 mM TCEP for equilibrating and washing the column (5 mL HisTrap FF

column (Cytiva, Marlborough, MA, USA)), whereas the same buffer but with a gradient from 10 to 250 mM imidazole was used for elution of CK2α'^{Cys336Ser}.

Expression of untagged CK2β$^{1-193}$, (His)$_6$-tagged CK2β$^{1-193}$, CK2β$^{1-193}$ tagged with Strep-tag® II and the Twin-Strep-tagged® proteins EGFP, mCherry, EGFP-CK2α$^{1-335}$, EGFP-CK2α'^{Cys336Ser} and mCherry-CK2β$^{1-193}$ was as follows: The plasmid containing the genetic information for the respective protein was transformed into *E. coli* BL21(DE3) bacteria using the heat shock method. For expression, single cell colonies were grown at 37 °C overnight in 200 mL LB medium (10 g/L tryptone, 5 g/L yeast extract, 10 g/L NaCl) containing either 100 µg/mL ampicillin (for untagged CK2β$^{1-193}$, CK2β$^{1-193}$ tagged with Strep-tag® II, EGFP, mCherry, EGFP-CK2α$^{1-335}$, EGFP-CK2α'^{Cys336Ser}, and mCherry-CK2β$^{1-193}$) or 75 µg/mL kanamycin (His-CK2β$^{1-193}$). The pre-culture was diluted 1:50 into 4x 1 L of fresh LB-Miller G medium (10 g/L tryptone, 5 g/L yeast extract, 10 g/L NaCl, 4 g/L glucose, 0.5 g/L $MgSO_4$, 5.4 g/L KH_2PO_4, 10.5 g/L K_2HPO_4, pH 7.0) containing 100 µg/mL ampicillin and 75 µg/mL kanamycin, respectively, and grown at 37 °C until an optical density (OD_{600}) of 0.4-0.8 was reached. Protein expression was induced by addition of isopropyl thio-D-galactopyranoside (IPTG) (final concentration of 0.5 mM) and cells were grown at 20 °C overnight. Cells were harvested by centrifugation (4 °C, 7000 -10,000 × g) and the cell pellet was stored at -80 °C until further use. For lysis, the cell pellet was thawed and suspended in the respective buffer (25 mM Tris, 300 mM NaCl, pH 8.5 for untagged CK2β$^{1-193}$; 20 mM Tris, 300 mM NaCl, pH 7.8 for His-tagged CK2β$^{1-193}$ and 100 mM Tris, 150 mM NaCl, pH 8.0 for all the other proteins). Cells expressing His-CK2β$^{1-193}$ or CK2β$^{1-193}$ tagged with Strep-tag® II were lysed for 1 h at 4 °C using 2 mg/mL lysozyme, 0.35 mg/mL PMSF, 0.2 mg/mL DNaseI and 50 mg/mL $MgSO_4$. Those cells expressing the fluorescently labelled proteins were lysed under the same conditions but with additional 50 µL of protease inhibitor cocktail (product no. ab201111, abcam, Cambridge, UK). Lysis of cells producing the untagged CK2β$^{1-193}$ was performed with 1 mg/mL lysozyme, 0.17 mg/mL PMSF, 0.1 mg/mL DNaseI and 25 mg/mL $MgSO_4$. The lysates were stored at -80 °C until further use.

Purification of untagged CK2β$^{1-193}$ was conducted as described by Raaf et al.[11]. Purification of proteins carrying the Strep-tag® II or the Twin-Strep-tag® was done by affinity chromatography on a 5 mL Strep-tactin XT superflow column (IBA-Lifesciences, Göttingen, Germany). After loading the supernatant onto the column and washing unbound material away with 100 mM Tris, 150 mM NaCl, pH 8.0, the protein was eluted using BXT-buffer (100 mM Tris, 150 mM NaCl, 1 mM EDTA and 50 mM biotin). For EGFP-CK2α$^{1-1335}$ and EGFP-CK2α'^{Cys336Ser}, a second purification step was added using a 1 mL HiTrap Heparin column (GE Healthcare, Düsseldorf, Germany). Equilibration of the column and loading of pre-purified proteins was done with a low salt buffer (25 mM Tris-HCl, 150 mM NaCl, pH 8.5). After washing the column with the same buffer, the purified proteins were eluted by a gradient containing an increasing proportion of high salt buffer (25 mM Tris-HCl, 1 M NaCl, pH 8.5). His-tagged CK2β$^{1-193}$ was purified using a 1 mL HiTrap Talon crude column (Cytiva, Marlborough, MA, USA). After equilibration and loading of the protein onto the column, unbound material was washed away using 20 mM Tris, 300 mM NaCl, 5 mM imidazole, pH 7.8. A gradient elution was done using the same buffer, which had a high imidazole content (300 mM).

All purified proteins in this work were concentrated and re-buffered in 25 mM Tris, 500 mM NaCl, pH 8.5 by ultracentrifugation using Merck Amicon™ Ultra-15 filters and stored at -80 °C.

2.3 Synthesis of Pc peptides

The cyclic peptides G(CRLYGXKIHGC)G (X = Phe, Pc, and X = 3-I-Phe, I-Pc) and the fluorescent FA probe CF-Ahx-Pc consisting of 5,6-carboxyfluorescein (CF) coupled via a 6-aminohexanoic acid linker (Ahx) to the N-terminus of Pc were synthesized by the group of Ines Neundorf (University of Cologne, Department of Chemistry and Biochemistry, Institute of Biochemistry, Germany) according to the procedure described by Lindenblatt et al.[1] Analytical data for the three peptides have previously been provided[1, 3, 12]. Stock solutions of Pc (10 mM), I-Pc (10 mM) and CF-Ahx-Pc (200 µM) was prepared in distilled water and stored at -80 °C in the dark.

2.4 High-throughput screening of compound library

Screening of a library of 67,584 compounds and initial hit validation was performed in assay buffer, i.e. 25 mM Tris, 500 mM NaCl, pH 8.5, 0.05% (v/v) Tween 20, with additional 2%(v/v) DMSO. For library screening, binding of 0.1 µM CF-Ahx-Pc to 1 µM CK2α$^{1-335}$ was followed in the presence of 20 µM of each compound. Positive and negative controls, i.e. 100% and 0% of signal, respectively, were determined with CF-Ahx-Pc in the presence and absence of CK2α$^{1-335}$. Experiments were conducted in Corning 384-well low flange black flat bottom polystyrene plates (product no. 3573); liquid handling was done with a BioTek MultiFlo Fx Multimode Dispenser and a Tecan Freedom EVO Workstation. All assays were performed in a total volume of 20.4 µL, with 0.4 µL of compound in DMSO being added to 10 µL of assay buffer followed by addition of 5 µL of 4× CF-Ahx-Pc and 5 µL of 4× CK2α$^{1-335}$ both dissolved in assay buffer. The assay mixture was incubated for 30 min at room temperature and the parallel and perpendicular fluorescence intensities were measured on a Perkin Elmer EnVision plate reader (excitation at 480/30 nm, emission at 535/40 nm). Fluorescence polarization (G factor = 0.95) was calculated according to the equation Polarization (mP) = 1000 * (S-G*P)/(S+G*P) with S and P being the parallel and the perpendicular fluorescence intensity, respectively.

Initial validation of primary hits was done by determination of their IC_{50} values as detailed in legend to Table S1. IC_{50} values of compounds, I, were calculated according to the four-parameter equation $Y = Bottom + ((TOP – BOTTOM)/(1 + ([I]/IC_{50})^{nH}))$. In this equation, TOP and BOTTOM correspond to the upper and lower plateau of the dose-response curve, respectively, whereas nH represents the Hill slope, i.e. the steepness of the curve. All calculations described in chapters 2.4, 2.5, and 2.7, were done using Microsoft Excel as part of Microsoft Office Professional Plus 2016 (Microsoft Cooperation, Redmond, WA, USA) and GrapPad Prism v10.2.3 for Windows (GraphPad Software, Boston, MA, USA). Cyclic pentapeptides identified in the initial library screening and selected for further characterisation were commercially obtained from ChemBridge Corporation (San Diego, CA, USA).

2.5 Fluorescence anisotropy assay

Fluorescence anisotropy (FA) assays following binding of CF-Ahx-Pc to both CK2α$^{1-335}$ and CK2α'^{Cys336Ser} and displacement of the probe from the proteins by competitors were conducted according to the procedure by Hochscherf et al.[3] and Lindenblatt et al.[1] with some alterations. FA was determined without any pre-incubation of probe and protein.

CK2α$^{1-335}$ was investigated in Corning 384-well low flange black flat bottom polystyrene plates (product no. 3573) in a total volume of 50 µL containing 0.1 µM CF-Ahx-Pc and 2-3 µM of protein. Displacement of probe by 0.1-300 µM Pc was followed using 25 mM Tris, 500 mM NaCl, pH 8.5, whereas 2% (v/v) of DMSO was added to the buffer for analysis of the cyclic peptides (0.1-100/300 µM). For CK2α'^{Cys336Ser}, black BRANDplates pureGrade 96-well plates with F-bottom wells (Brand, Wertheim, Germany) and a total volume of 200 µL were used. Displacement of 0.1 µM CF-Ahx-Pc from 30 µM of protein by 3-300 µM Pc was investigated in 25 mM Tris, 500 mM NaCl, pH 8.5. The dissociation constant of CF-Ahx-Pc (0.1 µM) on the two proteins was determined with 0.001-100 µM CK2α$^{1-335}$ and 0.003-100 µM CK2α'^{Cys336Ser}, respectively.

2.6 Native PAGE for analysis of protein-protein interactions

For native polyacrylamide gel electrophoresis (native PAGE) and sodium dodecyl sulphate-PAGE (SDS-PAGE) a 10% separating gel and a 4% stacking gel were used. For native PAGE, the stacking gel consisted of 0.125 M Tris, pH 6.8, and 10% (v/v) acrylamide-bisacrylamide, whereas the separating gel contained 0.375 M Tris, pH 8.8, and 10% (v/v) acrylamide-bisacrylamide. For SDS-PAGE, both gels had the same composition but contained additional 0.1% (m/v) SDS. Loading of the samples onto the gel was conducted using three parts of protein mixed with one part of loading buffer (native PAGE: 240 mM Tris-HCl pH 6.8, 40% (v/v) glycerine and 0.08% bromophenol blue; SDS-PAGE: the same buffer with addition of 8% (m/v) SDS and 2% (v/v) 2-mercaptoethanol). To analyse protein-protein interaction, proteins were prepared either separately or in combination to give a final concentration of 20 µM and incubated for 15 min at room temperature. Electrophoretic runs were conducted at 80 V,

300 mA, 30 W (native PAGE) or 120 V, 300 mA, 30 W (SDS-PAGE) in running buffer (native PAGE: 25 mM Tris, pH 8.3, 0.2 M glycine; SDS-PAGE: the same buffer with additional 0.5% (m/v) SDS). Fluorescence of native PAGE gels was visualized on an Invitrogen™ iBright™ FL1500 imaging system using both the green (λ_{ex} = 455-485 nm, λ_{em} = 508-557 nm) and red (λ_{ex} = 515-545 nm, λ_{em} = 568-617 nm) channels. In addition, gels were stained with colloidal Coomassie Brilliant Blue G250 and destained several times with 10% (v/v) aqueous ethanol containing 2% (v/v) phosphoric acid[13]. Afterwards, gels were scanned using a LI-COR Odyssey Fc Imager (700 nm-channel) and analysed with Image Studio software version 5.2.5.

2.7 Analysis of protein-protein interactions by FRET

2.7.1 General information

FRET-based assays were conducted at 30 °C in either Corning 384-well low flange black flat bottom polystyrene plates (product no. 3573, total assay volume: 50 µL) or black BRANDplates pureGrade 96-well plates with F-bottom wells (total assay volume: 200 µL). Experiments were performed on a Synergy™ 2 plate reader (BioTek, USA) using filters for excitation and emission in combination with a 50% mirror or an Infinite® M1000 Pro plate reader (Tecan Group, Switzerland) equipped with a monochromator. The following excitation and emission wavelengths were used on a Synergy™ 2 plate reader: donor channel (D) 485/25 nm, 528/20 nm; FRET channel (F) 485/25 nm, 620/40 nm; acceptor channel (A) 540/25 nm, 620/40 nm. The gain factor was automatically determined. Excitation and emission wavelengths on the Infinite® M1000 Pro plate reader were the same, except using a bandwidth of 10 nm and a gain factor of 100 or 110. If not stated otherwise, experiments were conducted in 25 mM Tris-HCl, 500 mM NaCl, pH 8.5 (assay buffer) containing both 2% (v/v) DMSO and 0.05% (v/v) Tween 20.

Fluorescence intensity was measured for the following assay compositions: either EGFP/EGFP-CK2α$^{1\text{-}335}$/EGFP-CK2α'^{Cys336Ser} (donor) or mCherry/mCherry-CK2β$^{1\text{-}193}$ (acceptor) alone (sample controls) or both in combination or blank wells containing assay buffer (with or without co-solvents). Values of fluorescence intensity obtained for blank wells were subtracted

from those of containing fluorescent protein(s). Due to spectral bleed through, corrected sample controls were needed to calculate the corrected FRET signal (FRETc) according to Youvan et al.[14] and Hochreiter et al.[15]. In this process, corrected fluorescence intensities with negative values were set to zero.

2.7.2 Dissociation constants (K_D) for binding of mCherry-CK2β$^{1-193}$ to EGFP-CK2α$^{1-335}$ or EGFP-CK2α'^{Cys336Ser}

Protein stocks kept on ice were freshly diluted with assay buffer containing either no additive or 2% (v/v) DMSO or 0.05% (v/v) Tween 20 or both co-solvents. A dilution series of mCherry-CK2β$^{1-193}$ or mCherry (final acceptor concentrations of 0.6 – 300 nM) was mixed with a constant amount of EGFP-CK2α$^{1-335}$, EGFP-CK2α'^{Cys336Ser} or EGFP (final donor concentration of 30 nM). In addition, sample controls containing the same concentrations of acceptor or donor were prepared to calculate FRETc. Three independent experiments were conducted in triplicate. Total assay volume was 200 µL for the following donor/acceptor combinations: EGFP-CK2α$^{1-335}$/mCherry-CK2β$^{1-193}$ (w/ DMSO, w/o DMSO and Tween) and EGFP-CK2α'^{Cys336Ser}/mCherry-CK2β$^{1-193}$ (w/ DMSO & Tween, w/ DMSO, w/ Tween, w/o DMSO and Tween). All the other donor/acceptor combinations shown in Figure 1a were investigated in a total volume of 50 µL. The dissociation constant, K_D, was calculated according to the equation

$$FRETc = FRETc_{free} + \frac{(FRETc_{bound} - FRETc_{free})([A]+[D]+K_D-\sqrt{([A]+[D]+K_D)^2-4[A][D]})}{2[D]},$$

with $FRETc_{free}$ and $FRETc_{bound}$ representing the FRETc values for unbound donor and donor completely bound to acceptor, respectively, while [A] and [D] being the concentrations of the acceptor and the donor, respectively.

2.7.3 Dissociation constants (K_i) of CK2 inhibitors

A dilution series of different concentrations of the inhibitor was pre-mixed with mCherry-CK2β$^{1-193}$ in assay buffer containing 2% (v/v) DMSO and 0.05% (v/v) Tween 20 to obtain 2× inhibitor and 2× acceptor. A volume of 25 µL of 2× EGFP-CK2α$^{1-335}$ or 2× EGFP-CK2α'^{Cys336Ser} in assay buffer with 2% (v/v) DMSO and 0.05% (v/v) Tween 20 was added to 25 µL of the pre-mix on a

384-well plate to obtain final acceptor and donor concentrations of 50 and 30 nM (1×), respectively. Sample controls were prepared by addition of 50 µL of either 1× donor or 1× acceptor to the well.

Untagged CK2β$^{1\text{-}193}$ and I-Pc were investigated with the same final acceptor and donor concentrations but in a total volume of 200 µL by adding 20 µL 10× inhibitor and 20 µL 10× mCherry-CK2β$^{1\text{-}193}$ to 140 µL of assay buffer. Fluorescence intensities were measured after addition of 20 µL 10× EGFP-CK2α$^{1\text{-}335}$ and corrected by those obtained for 200 µL 1× sample controls. Three to four individual experiments were conducted in duplicate to triplicate. To test for self-fluorescence of the inhibitors, a concentration of 100 µM of inhibitor was investigated in the absence of the fluorescent proteins. In case of self-fluorescence as found for tagged and untagged CK2β$^{1\text{-}193}$ as well as for Pc and I-Pc, the fluorescence intensities were determined as described above without addition of either donor (tagged CK2β$^{1\text{-}193}$ and Pc) or donor and acceptor (untagged CK2β$^{1\text{-}193}$ and I-Pc) and used to correct fluorescence intensities obtained in the presence of both donor and acceptor. As the compounds from the library screen and their derivatives did not show any self-fluorescence at 100 µM, measured fluorescence intensities were not further corrected in this regard. IC_{50} values were determined by means of the four-parameter equation and used to calculate K_i values according to Nikolovska-Coleska et al.[2]. Herein, K_D values determined for several preparations of acceptor and donor proteins were applied.

2.7.4 Disruption of *in situ*-formed EGFP-CK2α$^{1\text{-}335}$/mCherry-CK2β$^{1\text{-}193}$ complexes

A mixture of EGFP-CK2α$^{1\text{-}335}$ and mCherry-CK2β$^{1\text{-}193}$ in assay buffer containing 2% (v/v) DMSO and 0.05% (v/v) Tween 20 (37.5 nM each in a total volume of 640 µL (for measurements shown in Figure 3d) or 1440 µL for IC_{50} determinations (Figures 3e,f, S6a,b) was incubated for 30 min at 30 °C to form complexes. As a control, either EGFP-CK2α$^{1\text{-}335}$ or mCherry-CK2β$^{1\text{-}193}$ alone in the same buffer (75 nM each in a total volume of 320 µL (for measurements shown in Figure 3d) or 800 µL for IC_{50} determinations (Figures 3e,f, S6a,b)) was incubated for 30 min at 30 °C. A volume of 10 µL of either 5× inhibitor in assay buffer with

2% (v/v) DMSO and 0.05% (v/v) Tween 20 (final concentration of 100 µM or 0.024-200 µM for IC_{50} determination) or the same buffer without inhibitor was pipetted into a 384 well plate. A volume of 40 µL of the pre-formed CK2 complexes or 20 µL of each of the two CK2 subunits (final concentration of 30 nM each) was added and fluorescence intensities were measured as described above immediately after addition of the proteins or after 15 min and 30 min, respectively. To correct for self-fluorescence, as it is the case for Pc, fluorescence intensities were determined without addition of either donor or acceptor and used to correct fluorescence intensities obtained in the presence of both donor and acceptor. To correct for spectral bleed-through, sample controls with 50 µL of 1× EGFP-CK2α$^{1-335}$ (30 nM) or 1× mCherry-CK2β$^{1-193}$ (30 nM) were analysed. Three to four separate experiments were performed in duplicate. Relative subunit binding for measurements shown in Figure 3d was calculated using the FRETc values obtained without compound as 100%. IC_{50} values were determined by means of the four-parameter equation.

2.8 CK2-catalyzed substrate phosphorylation

Kinase activity of CK2α and CK2$\alpha_2\beta_2$ holoenzyme was quantified by analysing phosphorylation of the peptides RRRDDDSDDD and eIF2β$^{1-22}$ by γ-^{32}P-ATP as previously described by Lindenblatt et al.[1] with some alterations.

For experiments with the individual CK2 subunits, full-length CK2α and full-length CK2β (90 nM each) were investigated in a total volume of 50 µL. To a volume of 18 µL of 4.4 pmol CK2α in kinase buffer (50 mM Tris-HCl, pH 7.5, 150 mM NaCl, 5 mM $MgCl_2$, 1 mM DTT), a volume of 1 µL of 5 mM inhibitor in DMSO or 1 µL of 0.05 mM SGC-CK2-1 in DMSO (final concentrations: 100 µM for compounds **12**, **14** and **15,** 1 µM for SGC-CK2-1) was added. After 1-2 min of incubation at room temperature, a volume of 1 µL of 4.4 pmol CK2β was added. The reaction was started by addition of 30 µL of a mixture of the substrate peptides (0.19 mM RRRDDDSDDD or eIF2β$^{1-22}$) and ATP (20 µCi mL^{-1} γ-^{32}P-ATP and 50 µM ATP) in assay buffer (25 mM Tris·HCl, pH 8.5, 150 mM NaCl, 5 mM $MgCl_2$, 1 mM DTT) either immediately or after

10 min of pre-incubation at room temperature. For experiments with CK2 holoenzyme, a final concentration of 10 nM CK2$\alpha_2\beta_2$ was used.

2.9 Crystallization and crystal structure determination

All protein crystallization experiments were performed using the sitting drop variant of the vapour diffusion method. For CK2α'^{Cys336Ser}, crystallization conditions to provide atomic-resolution structures are established[16]; in the underlying triclinic crystal packing, however, the CK2β interface is not accessible for small molecule ligands. Therefore, we adapted a CK2α'^{Cys336Ser} crystallization strategy recently described by Werner et al.[10] and based on a slight reduction of the LiCl concentration. This procedure provided an alternative monoclinic crystal packing in which one of two CK2α'^{Cys336Ser} protomers is no longer blocked at the CK2β interface. In this way, we could successfully co-crystallize CK2α'^{Cys336Ser} with compound **12** (but not with compound **15**).

The optimal reservoir composition for the crystallization of the CK2α'^{Cys336Ser}/**12** complex was 28 % (w/v) PEG 6000, 810 mM LiCl, 100 mM Tris-HCl, pH 8.5. CK2α'^{Cys336Ser} with a concentration of 5 mg/mL was mixed in a 1:10 ratio with 10 mM compound **12** in DMSO. This mixture was incubated on ice for 30 min. Afterwards, any precipitate was removed by centrifugation (20000 × g, room temperature, 2 min). Using sitting-drop crystallization plates, the CK2α'^{Cys336Ser}/**12** solution was mixed in a 2:1 ratio with reservoir solution and pre-incubated at 20 °C for one week. To induce crystal formation, micro seeds from another set of monoclinic CK2α'^{Cys336Ser} crystals were added. This led to crystals that were subsequently used as macro seeds. The resulting crystals were cryo-protected by transferring them to a mixture of 70% reservoir solution and 30% ethylene glycol and afterwards flash-frozen in liquid nitrogen. X-ray diffraction data were collected at beamline ID30B[5] of the ESRF.

For co-crystallization of CK2α$^{1\text{-}335}$ with compounds **12** and **15**, no known conditions were adapted; rather, we tested several commercially available crystallization screens, different protein-to-ligand ratios and temperatures as well as micro seeding. The final hit used for any subsequent crystallization experiment with CK2α$^{1\text{-}335}$ and either compound **12** or **15** was

reagent 16 (1.5 M lithium sulphate, 0.1 M Na-HEPES, pH 7.5) of the Crystal screen from Hampton Research. After optimization, the best procedure was as follows: 7 mg/ml $CK2\alpha^{1-335}$ was mixed in a 1:40 ratio with a 20 mM solution of compound **12** or **15** in DMSO. The $CK2\alpha^{1-335}$/ligand mixture was incubated at 20 °C for 30 min. After removing precipitate by centrifugation (20000 $\times$ g, 2 min, 20 °C), the $CK2\alpha^{1-335}$/ligand solution was mixed in a 2:1 ratio with the reservoir solution (1.5 M lithium sulphate, 0.1 M Na-HEPES, pH 7.5) in a 24-well sitting drop plate. The drop was pre-equilibrated against 700 µL reservoir solution. $CK2\alpha^{1-335}$/**15** crystals formed spontaneously. In the case of $CK2\alpha^{1-335}$/**12**, crystals growth had to be induced by micro seeding using a seed suspension prepared as 1:1000 dilution of one $CK2\alpha^{1-335}$/**15** crystal crushed in 100 µl reservoir solution. Crystals were harvested after 3 weeks (with compound **12**) or 2 months (with compound **15**) by flash-freezing in liquid nitrogen without any additional cryo-protection. X-ray diffraction data of the $CK2\alpha^{1-335}$ crystals were collected at beamlines ID30A-3 (MASSIF-3)[6] or ID23-1[7] of the ESRF.

All X-ray diffraction data were processed using the autoPROC pipeline[17]. This includes indexing and integration by XDS[18], symmetry analysis by POINTLESS[19] and scaling by AIMLESS[20] from the CCP4 software suite[21]. Finally, STARANISO[8] was applied for anisotropy analysis. It provided ellipsoidal diffraction data sets that were applied for structure determination by molecular replacement using PHASER[22] within Phenix[23]. Structure optimization were performed by manual modelling with Coot[24] alternating with computational refinement with Phenix[25]. The novel ligands **12** and **15** were parametrized with eLBOW[26].

3. References

1. D. Lindenblatt, M. Horn, C. Götz, K. Niefind, I. Neundorf and M. Pietsch, ChemMedChem, 2019, 14, 833-841.
2. Z. Nikolovska-Coleska, R. Wang, X. Fang, H. Pan, Y. Tomita, P. Li, P. P. Roller, K. Krajewski, N. G. Saito, J. A. Stuckey and S. Wang, Anal Biochem, 2004, 332, 261-273.
3. J. Hochscherf, D. Lindenblatt, M. Steinkrüger, E. Yoo, Ö. Ulucan, S. Herzig, O. G. Issinger, V. Helms, C. Götz, I. Neundorf, K. Niefind and M. Pietsch, Anal Biochem, 2015, 468, 4-14.
4. C. I. Wells, D. H. Drewry, J. E. Pickett, A. Tjaden, A. Krämer, S. Müller, L. Gyenis, D. Menyhart, D. W. Litchfield, S. Knapp and A. D. Axtman, Cell Chem Biol, 2021, 28, 546-558.e510.
5. A. A. McCarthy, R. Barrett, A. Beteva, H. Caserotto, F. Dobias, F. Felisaz, T. Giraud, M. Guijarro, R. Janocha, A. Khadrouche, M. Lentini, G. A. Leonard, M. Lopez Marrero, S. Malbet-Monaco, S. McSweeney, D. Nurizzo, G. Papp, C. Rossi, J. Sinoir, ... and C. Mueller-Dieckmann, J Synchrotron Radiat, 2018, 25, 1249-1260.
6. D. von Stetten, P. Carpentier, D. Flot, A. Beteva, H. Caserotto, F. Dobias, M. Guijarro, T. Giraud, M. Lentini, S. McSweeney, A. Royant, S. Petitdemange, J. Sinoir, J. Surr, O. Svensson, P. Theveneau, G. A. Leonard and C. Mueller-Dieckmann, J Synchrotron Radiat, 2020, 27, 844-851.
7. D. Nurizzo, T. Mairs, M. Guijarro, V. Rey, J. Meyer, P. Fajardo, J. Chavanne, J. C. Biasci, S. McSweeney and E. Mitchell, J Synchrotron Radiat, 2006, 13, 227-238.
8. I. J. Tickle, C. Flensburg, P. Keller, W. Paciorek, A. Sharff, C. Vonrhein and G. Bricogne (2016). STARANISO. Cambridge, United Kingdom: Global Phasing Ltd..
9. C. Werner, A. Gast, D. Lindenblatt, A. Nickelsen, K. Niefind, J. Jose and J. Hochscherf, Front Mol Biosci, 2022, 9, 831693.
10. C. Werner, D. Lindenblatt, K. Viht, A. Uri and K. Niefind, Kinases and Phosphatases, 2023, 1, 306-322.
11. J. Raaf, E. Brunstein, O.-G. Issinger and K. Niefind, Protein Sci, 2008, 17, 2180-2186.
12. J. Raaf, B. Guerra, I. Neundorf, B. Bopp, O. G. Issinger, J. Jose, M. Pietsch and K. Niefind, ACS Chem Biol, 2013, 8, 901-907.
13. Kang, D. H., Y. S. Gho, M. K. Suh and C. H. Kang, Bull. Korean Chem. Soc., 2002, 23, 1511-1512.

14. D. C. Youvan, C. M. Silva, E. J. Bylina, W. J. Coleman, M. R. Dilworth and M. M. Yang, Biotechnology et alia, 1997, 3, 1-18.

15. B. Hochreiter, M. Kunze, B. Moser and J. A. Schmid, Sci Rep, 2019, 9, 8233.

16. D. Lindenblatt, A. Nickelsen, V. M. Applegate, J. Hochscherf, B. Witulski, Z. Bouaziz, C. Marminon, M. Bretner, M. Le Borgne, J. Jose and K. Niefind, ACS Omega, 2019, 4, 5471-5478.

17. C. Vonrhein, C. Flensburg, P. Keller, A. Sharff, O. Smart, W. Paciorek, T. Womack and G. Bricogne, Acta Crystallogr D Biol Crystallogr, 2011, 67, 293-302.

18. W. Kabsch, Acta Crystallogr D Biol Crystallogr, 2010, 66, 125-132.

19. P. Evans, Acta Crystallogr D Biol Crystallogr, 2006, 62, 72-82.

20. P. R. Evans and G. N. Murshudov, Acta Crystallogr D Biol Crystallogr, 2013, 69, 1204-1214.

21. M. D. Winn, C. C. Ballard, K. D. Cowtan, E. J. Dodson, P. Emsley, P. R. Evans, R. M. Keegan, E. B. Krissinel, A. G. Leslie, A. McCoy, S. J. McNicholas, G. N. Murshudov, N. S. Pannu, E. A. Potterton, H. R. Powell, R. J. Read, A. Vagin and K. S. Wilson, Acta Crystallogr D Biol Crystallogr, 2011, 67, 235-242.

22. A. J. McCoy, R. W. Grosse-Kunstleve, P. D. Adams, M. D. Winn, L. C. Storoni and R. J. Read, J Appl Crystallogr, 2007, 40, 658-674.

23. P. D. Adams, P. V. Afonine, G. Bunkóczi, V. B. Chen, I. W. Davis, N. Echols, J. J. Headd, L. W. Hung, G. J. Kapral, R. W. Grosse-Kunstleve, A. J. McCoy, N. W. Moriarty, R. Oeffner, R. J. Read, D. C. Richardson, J. S. Richardson, T. C. Terwilliger and P. H. Zwart, Acta Crystallogr D Biol Crystallogr, 2010, 66, 213-221.

24. P. Emsley, B. Lohkamp, W. G. Scott and K. Cowtan, Acta Crystallogr D Biol Crystallogr, 2010, 66, 486-501.

25. P. V. Afonine, R. W. Grosse-Kunstleve, N. Echols, J. J. Headd, N. W. Moriarty, M. Mustyakimov, T. C. Terwilliger, A. Urzhumtsev, P. H. Zwart and P. D. Adams, Acta Crystallogr D Biol Crystallogr, 2012, 68, 352-367.

26. N. W. Moriarty, R. W. Grosse-Kunstleve and P. D. Adams, Acta Crystallogr D Biol Crystallogr, 2009, 65, 1074-1080.
